# Rapid adaptation to invasive predators overwhelms natural gradients of intraspecific variation

**DOI:** 10.1038/s41467-020-17406-y

**Published:** 2020-07-17

**Authors:** Andrea Melotto, Raoul Manenti, Gentile Francesco Ficetola

**Affiliations:** 1 Department of Environmental Science and Policy, Università degli Studi di Milano, Via Celoria 26, Milano, 20133 Italy; 2 Centre of Excellence for Invasion Biology, Department of Botany and Zoology, Stellenbosch University, Stellenbosch, Western Cape South Africa; 3 Univ. Grenoble Alpes, Univ. Savoie Mont Blanc, CNRS, LECA - Laboratoire d’Écologie Alpine, F-38000 Grenoble, France

**Keywords:** Community ecology, Evolutionary ecology, Invasive species, Herpetology

## Abstract

Invasive predators can exert strong selection on native populations. If selection is strong enough, populations could lose the phenotypic variation caused by adaptation to heterogeneous environments. We compare frog tadpoles prior to and 14 years following invasion by crayfish. Prior to the invasion, populations differed in their intrinsic developmental rate, with tadpoles from cold areas reaching metamorphosis sooner than those from warm areas. Following the invasion, tadpoles from invaded populations develop faster than those from non-invaded populations. This ontogenetic shift overwhelmed the intraspecific variation between populations in a few generations, to the point where invaded populations develop at a similar rate regardless of climate. Rapid development can have costs, as fast-developing froglets have a smaller body size and poorer jumping performance, but compensatory growth counteracts some costs of development acceleration. Strong selection by invasive species can disrupt local adaptations by dampening intraspecific phenotypic variation, with complex consequences on lifetime fitness.

## Abstract (web summary)

Invasive species can influence the evolution of natives. Here, authors use common garden experiments to show that invasive predatory crayfish have homogenized the developmental timing of a native frog by selecting for more rapid development to avoid predation.

## Introduction

Invasive alien species threaten biodiversity at the global scale^1–3^. The decline of native species due to invasive organisms is often attributed to a lack of common evolutionary history, which can determine the absence of effective responses^4,5^. On the other hand, the abrupt selective pressure exerted by invasives can promote the rapid expression of traits improving fitness during interactions between native and alien species. Such responses include phenotypic plasticity (e.g. developmental or behavioural plasticity), and may lead to prompt adaptations in native species^6–11^. Nevertheless, native species often inhabit heterogeneous environments, and populations exposed to diverging selective pressures by natural gradients can show local adaptations, which allow them to cope with different environmental challenges^12^. The new selective forces exerted by invasive species are expected to interfere with the pressures imposed by the extant environmental context^13^, thus the effectiveness and long-term consequences of evolutionary responses to invasive species remain difficult to predict^14^. Despite the growing literature on the evolutionary consequences of biological invasions, few studies have considered how selective pressures imposed by invasive species interfere with pre-existing patterns of local adaptations and environmental heterogeneity of native populations^15,16^. This is likely a result of the complexity of disentangling multiple selective forces. Long-term studies, comparing species responses before and after the introduction of invasive species, can provide key insights on how interactions between multiple selective forces shape evolutionary trajectories.

Development time is a critical life history trait of ectotherms that can show both plastic and canalized variation in response to environmental pressures. For instance, ectothermic metabolism slows down with cold temperatures and developmental rate is typically reduced as a result. Therefore, populations living in cold environments often evolve faster intrinsic development time, which allows them to partially counteract the dampening effect of low temperature (i.e. counter-gradient variation^17,18^). However, predator presence often selects for a fast development time, which can reduce the prey’s exposure to predators^19–21^. As development time can respond to multiple selective forces with complex patterns, it is an excellent trait to evaluate the interplay between natural selective gradients and the pressure by invasive species.

Complex life cycles, with larvae strongly different from adults, are widespread across animals. In organisms with complex life cycles, the analysis of life history traits is complicated by trade-offs in trait-expression between stages, as responses to selective pressures experienced early on can cause carry-over effects in later stages^22^. For instance, accelerated development in larvae can reduce the time available to harvest trophic resources, thereby limiting investment in morphological structures (e.g. body size, muscles and fat reserves), with potential fitness consequences after metamorphosis^22,23^. On the other hand, compensatory mechanisms are also possible, limiting the lifetime consequences of plastic and adaptive responses^24^.

**Fig. 1:**
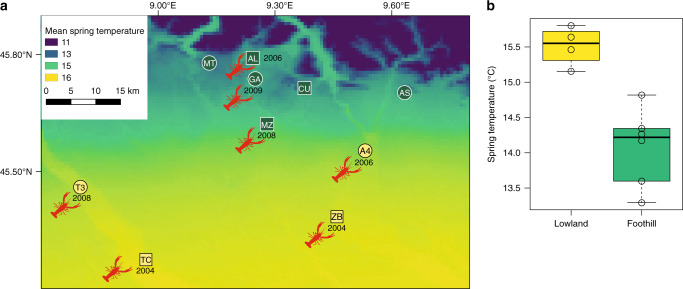
Climatic regime and invasion status in the study area. **a** Location of study populations. Squares represent frog populations analysed before the crayfish invasion (2003), whereas circles indicate populations added for analyses performed after the crayfish invasion (2017). Invaded populations are labelled by a crayfish and, for each of them, we report the year of first detection of the crayfish. Foothill populations are in dark green; lowland populations are in pale yellow. The background map also shows average spring temperature, from warmer lowlands to colder foothills. **b** Boxplot representing differences in spring temperature between the six foothill and the four lowland breeding sites (values of mean spring temperature, *n* = 10 localities; two-tailed student’s *t* test: *t*_7_ = 3.97; *P* = 0.005). Box limits represent the first and the third quartiles, the central line is the median, the whiskers represent the extreme values. Source data are provided as a Source data file.

Populations of native amphibians exposed to divergent environmental gradients often show strong variation in developmental rate. For instance, the Italian agile frog, *Rana latastei*, inhabits both lowland and foothill sites, where tadpoles are exposed to different climatic regimes (lower temperatures in the foothills; Fig. 1b). When reared within the same temperature conditions, individuals showed significant differences in intrinsic development time across populations, with tadpoles from the colder foothills reaching metamorphosis earlier, as expected under a pattern of counter-gradient variation^25^. Such adaptive variability was recorded in 2003, immediately before the invasion by the American red swamp crayfish (*Procambarus clarkii*). This generalist predator has been regarded as among the “100 worst” alien species in the world^26,27^ and can impose dramatic predation pressure on aquatic amphibians, especially on their larvae^28–30^. Nevertheless, amphibians can show both plastic and rapid evolutionary responses to recent environmental changes that can help them to withstand these novel challenges^6,31,32^.

Here we evaluate whether the selective pressure exerted by an invasive predator can produce rapid adaptation in developmental rate of the Italian agile frog, and assess how invasive species altered the pattern of intra-specific variation in populations living along an environmental gradient. By repeating common-garden analyses of intrinsic development time before and after the invasion (i.e. over 14 years), we assess how two distinct selective forces (i.e. climatic heterogeneity and novel predators) influence adaptive variation across space. We predict that selection favours faster development of tadpoles after the crayfish invasion, and that this novel pressure will decrease the ontogenetic variation between populations from different climatic regimes. Our study shows that the heavy selection by invasive predators causes the loss of intraspecific variation among native populations, with the novel selective force overwhelming the role of climate as a key driver of development rate in frog populations. Changes in development time caused by the invasive crayfish have profound consequences on post-metamorphic traits and froglet performance, even though compensating mechanisms alleviating these effects are also present.

## Results

### Differences between populations before crayfish invasion

**Fig. 2:**
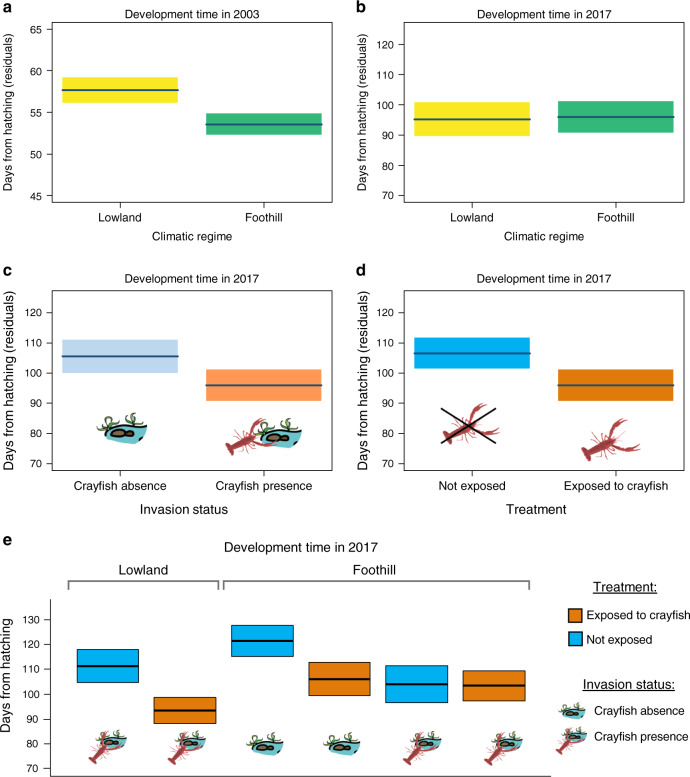
Factors affecting development time (days from hatching) of Italian agile frog, *Rana latastei*, tadpoles. Conditional partial residual plots showing the effect of **a** climatic regime before the crayfish invasion; **b** climatic regime after the crayfish invasion; **c** invasion status of the population and **d** crayfish exposure during rearing on development time (calculated as days from hatching to metamorphosis). Bold blue lines represent the average value obtained using mixed models, while shaded areas are 95% confidence bands. **e** Boxplots showing the simultaneous effect of climatic regime, invasion status and experimental crayfish exposure on development time of tadpoles. Orange boxes represent tadpoles exposed to the crayfish during rearing, while blue boxes represent individuals not exposed. Images of ponds with crayfish represent invaded populations, while those with no crayfish represent the uninvaded ones. All the lowland breeding sites are colonized by the invasive crayfish. The black line represents the mean value while the box limits are ±2 standard errors. In panel (**a**), *n* = 180 tadpoles; in panels (**b**–**e**), *n* = 169 tadpoles. Source data are provided as a Source data file.

A common-garden experiment was performed before the crayfish invasion to measure the differences in development time between frogs from diverging climatic regimes (three foothill and two lowland populations; Fig. 1a). Prior to the invasion, tadpoles from foothill populations showed faster intrinsic development times than those originating from lowland populations (mixed models, *p* < 0.001; Table 1a) and under common environmental conditions foothill tadpoles reached metamorphosis on average 4.1 days earlier (Fig. 2a). This likely occurred because spring temperature was on average 1.4 °C colder in foothills, compared with lowland sites (Fig. 1b). The fast intrinsic development time in foothill populations was therefore interpreted as an evolutionary adaptation to the cold climate (counter-gradient variation)^25^.

**Table 1:** Factors determining development time of agile frogs, before (a) and after (b) crayfish invasion: results of linear mixed models (two-sided F tests).

Fixed effects	F	df	*P*
(a) 2003: before crayfish invasion			
Climatic regime	17.52	1, 16.3	**<0.001**
No. of siblings	1.48	1, 28.4	0.234
(b) 2017: after crayfish invasion			
Climatic regime	0.02	1, 52.3	0.879
Invasion status	9.72	1, 51.6	**0.003**
Crayfish exposure	30.13	1, 163.9	**<0.001**
No. of siblings	7.06	1, 145.5	**0.009**
Invasion status × crayfish exposure	6.30	1, 162.9	**0.013**
Climatic regime × crayfish exposure	12.32	1, 161.4	**<0.001**

All models included the climatic regime (lowland vs. foothill) as a fixed factor. Experiments performed in 2017 included two additional factors: invasion status (crayfish invasion in the wetland of origin) and crayfish exposure (presence of crayfish in the container during rearing). All models included the *N* of siblings in the container, to take into account potential effects of tadpole density. Sample size was not identical among treatments; thus, degrees of freedom can be not integer. Significant effects are in bold. In the 2003 experiment, *n* = 180 tadpoles; in the 2017 experiment, *n* = 169 tadpoles.

### Crayfish invasion now drives developmental variation

In the early 2000s, the invasive crayfish was first detected in southern Lombardy and then it spread northward^33^. The local climate did not show any evident change in temperature or precipitation during 2000–2017 (both *p* > 0.15), and the climatic differences existing between foothills and lowlands remained consistent (Supplementary Note 1; Supplementary Table 1). To assess how the new selective pressure posed by the invasive crayfish may have affected frogs, and how this can interact with the extant selective forces, we repeated the analysis of larval development time 14 years after the onset of the invasion. Among the nine populations analysed in 2017, all the lowland populations and half of the foothill populations had been invaded between 2004 and 2009, while the crayfish is still absent in the remaining foothill populations (Fig. 1a). To test the possibility of predator-induced phenotypic plasticity, in this post-invasion experiment, tadpoles were also randomly assigned to two treatments: absence vs. non-lethal presence of the invasive crayfish in the container during rearing.

After the crayfish invasion, differences in intrinsic development time between foothill and lowland populations were no longer significant (*p* = 0.879; Table 1b, Fig. 2b), while we did find significant differences in development time in response to the invasive crayfish. In 2017, intrinsic development time was significantly faster in tadpoles from invaded populations compared to those from uninvaded populations (*p* = 0.003; Fig. 2c), suggesting that the novel predation pressure had become a dominant driver of the length of larval stage. Furthermore, development time was faster in tadpoles exposed to crayfish (*p* < 0.001; Fig. 2d), suggesting that tadpoles activate a plastic anti-predatory response by shifting their developmental time. We also found significant interactions between crayfish exposure and invasion status (*p* = 0.013), and between crayfish exposure and climatic regime (*p* < 0.001). Tadpoles exposed to crayfish accelerated their development more if they originated from lowland populations, and if they originated from populations invaded by the crayfish (Fig. 2e). Finally, development time was significantly faster in containers where few tadpoles survived until metamorphosis (*p* = 0.009), in agreement with known effects of intraspecific competition^34^. In the 2017 experiment, the total development time was generally longer than in the 2003 experiment (e.g. Fig. 2a, b), possibly because tadpoles were exposed to diel temperature fluctuations (ref. ^35^; see Supplementary Note 2). This is supported by the observation that total development time in the 2017 experiment was comparable to the development time generally observed in nature (refs. ^25,36^; Supplementary Note 2), although other potential causes of these differences cannot be excluded.

### Accelerated development affects post-metamorphic traits

**Fig. 3:**
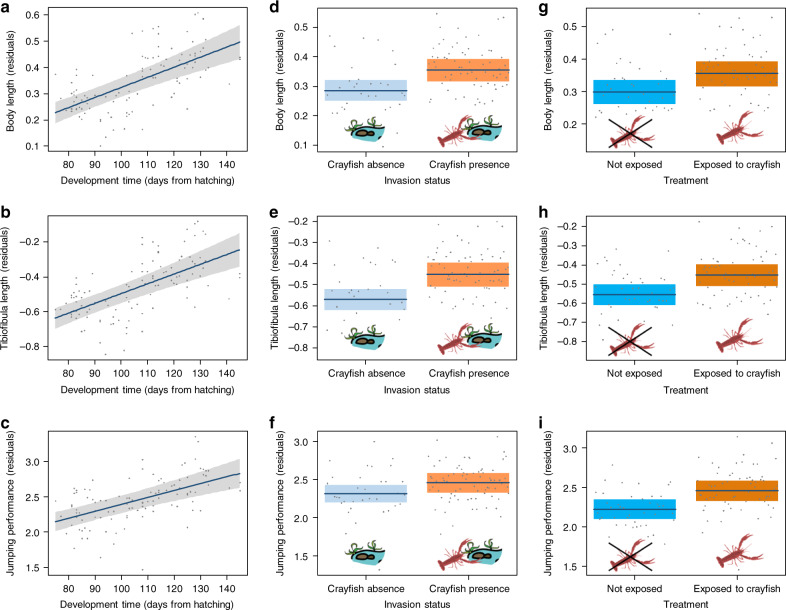
Factors affecting post-metamorphic traits of froglets. Plots are conditional partial residual plots showing the influence on froglet body length, tibiofibula length and maximum jumping distance of development time (**a**–**c**), invasion status of the population (**d**–**f**) and crayfish exposure during rearing (**g**–**i**). Bold blue lines represent the average value obtained using mixed models, while shaded areas represent 95% confidence bands. *n* = 110 froglets. Source data are provided as a Source data file.

To assess potential carry-over effects of anti-predator strategies, we evaluated whether fast larval development affects the variation of multiple traits after metamorphosis (body length, tibiofibula length, and maximum jumping distance). Faster larval development led to froglets with a smaller body size (F_1, 101.3_ = 37.91; *p* < 0.001; Fig. 3a) and shorter hind limbs (F_1, 96.6_ = 26.38; *p* < 0.001; Fig. 3b). Froglets with shorter development times also showed reduced locomotor performance, as maximum jumping distance increased in froglets that took longer to reach metamorphosis (F_1, 105.5_ = 20.62; *p* < 0.001; Fig. 3c). This occurred because froglets with longer tibiofibula were able to perform longer jumps (F_1, 99.3_ = 98.07; *p* < 0.001), while after taking into account the effect of tibiofibula length, the relationship between development time and jumping performance was no longer significant (F_1, 88.2_ = 0.63; *p* = 0.428).

Accounting for differences in development time, tadpoles exposed to crayfish showed larger body sizes (*p* = 0.023), longer legs (*p* = 0.007), and better jumping performance (*p* = 0.015) (Table 2a–c; Fig. 3g–i). Similarly, tadpoles from invaded populations showed larger body sizes (*p* = 0.002), longer legs (*p* < 0.001), and performed longer jumps (*p* = 0.042) when compared to tadpoles from uninvaded populations (Table 2a–c; Fig. 3d–f). Furthermore, larval density (i.e. the number of individuals within a container) always negatively affected all of the post-metamorphic traits (Table 2a–c) and tadpoles from foothill populations showed larger body sizes (*p* = 0.017). All the effects of treatment and tadpole origin on post-metamorphic traits, however, were non-significant if development time was not taken into account (all *p* > 0.05; Table 2d–f).

**Table 2:** Effect of treatment, invasion status, climatic regime and number of siblings on post-metamorphic traits of Italian agile frogs: results of linear mixed models (two-sided F tests without multiple test corrections) including development time as fixed factor in the model (a–c) and results excluding development time from the model (d–f).

Post-metamorphic trait	Fixed effects	F	df	*P*	*R*^2^_m_	*R*^2^_c_
Analyses including development time as covariate						
(a) Body size	Climatic regime	5.189	1, 99.5	**0.017**		
	Invasion status	9.79	1, 103.0	**0.002**		
	Crayfish exposure	7.42	1, 9.2	**0.023**	0.35	0.35
	No. of siblings	7.44	1, 102.9	**0.007**		
	Development time	50.38	1, 100.5	**<0.001**		
(b) Tibiofibula length	Climatic regime	1. 77	1, 100.6	0.187	0.33	0.33
	Invasion status	12. 71	1, 104.2	**<0.001**		
	Crayfish exposure	12.06	1, 9.4	**0.007**		
	No. of siblings	10.29	1, 104.4	**0.002**		
	Development time	47.84	1, 101.6	**<0.001**		
(c) Max jumping distance	Climatic regime	1.39	1, 101.5	0.242		
	Invasion status	4.22	1, 107.7	**0.042**		
	Crayfish exposure	10.20	1, 7.2	**0.015**	0.27	0.30
	No. of siblings	12.11	1, 106.8	**<0.001**		
	Development time	35.034	1, 102.4	**<0.001**		
Analyses not including development time as covariate						
(d) Body size	Climatic regime	2. 94	1, 104.0	0.089		
	Invasion status	0.77	1, 104.0	0.382		
	Crayfish exposure	0.12	1, 104.0	0.730	0.03	0.03
	No. of siblings	0.11	1, 104.0	0.740		
(e) Tibiofibula length	Climatic regime	0.66	1, 105.0	0.417		
	Invasion status	1.55	1, 105.0	0.216		
	Crayfish exposure	0.90	1, 105.0	0.344	0.02	0.02
	No. of siblings	0.65	1, 105.0	0.420		
(f) Max jumping distance	Climatic regime	0.71	1, 101.3	0.402		
	Invasion status	0.08	1, 108.0	0.782		
	Crayfish exposure	2.07	1, 5.8	0.202	0.04	0.06
	No. of siblings	1.82	1, 108.0	0.180		

For all tests we report both marginal and conditional determination coefficients (*R*^2^_m_ and *R*^2^_m_, respectively^74^). Significant effects are in bold. *n* = 110 tadpoles.

**Fig. 4:**
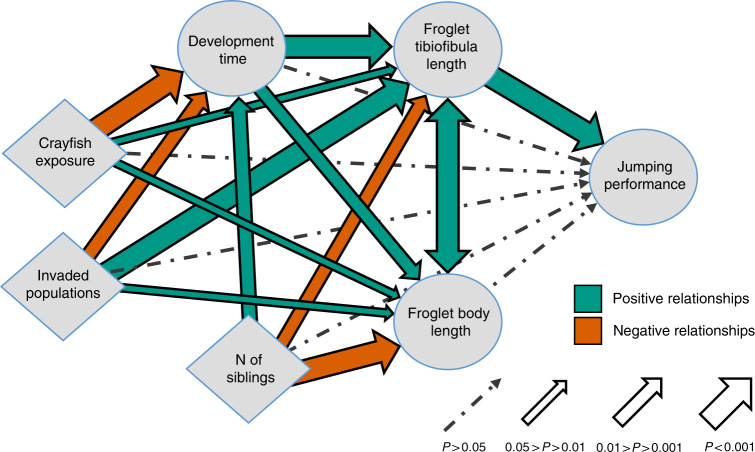
Structural equation model showing the overall effects of invasive crayfish on tadpole development. Diamonds represents fixed effects, while circles indicate dependent variables. Significance of relationships is indicated with arrows, and was calculated from SEM using two-sided *z* statistics. Positive relationships are in green, while negative relationships are in red. The number of individuals per container was included as a covariate, to take into account the effects of density. See Supplementary Table 2 for coefficients and exact significance values of the paths.

Finally, we used structural equation models to evaluate the overall consequences of the invasive crayfish (considering both local adaptations and plasticity) on post-metamorphic traits, and how these shifts are mediated by developmental acceleration (Fig. 4). Both the crayfish invasion at breeding sites and exposure to crayfish during development determined a short larval development time. This, in turn, negatively affected both body and tibiofibula length of froglets which resulted in poorer jumping performance (Fig. 4). These effects, however, came with positive relationships between post-metamorphic traits and both crayfish exposure and invasion status, which partly compensated the costs of the shorter development induced by the crayfish (Fig. 4; see also Supplementary Table 2).

## Discussion

Alien species can exert severe selective pressure within invaded ecosystems, generating eco-evolutionary interactions and fostering both plasticity and rapid evolution in native species^14,37^. The novel predation pressure imposed by the invasive crayfish in this study was strong enough to disrupt the pre-existing local adaptation in frogs to the regional climatic regime in a period of just 8–14 years (3–6 generations), by causing a faster development in tadpoles from invaded populations. This developmental acceleration may have long-term associated costs, as the shorter larval duration resulted in poorer conditions at metamorphosis, even though compensatory growth could limit these impacts.

Temperature strongly affects amphibian ontogeny^31^ and spatial variation in climate can determine adaptive shifts at both the species- and population-level^6,12,17^. Before the crayfish invasion, Italian agile frog populations showed significant divergence for intrinsic development time (Fig. 2a) across an existing temperature gradient (colder climate in foothills; Fig. 1b). Such a pattern suggests that climate was a major selective force and counter-gradient selection caused clear local adaptations, with foothill populations showing faster development to counteract environmental constraints. Starting from 2004, however, the invasive crayfish spread across the study area^33^, successfully colonizing breeding sites and representing an unprecedented selective pressure for these frogs. The invasion was particularly intense in lowlands^38^, where nearly all the breeding sites are now colonized by crayfish. As a result of the crayfish invasion, differences in development time between foothill and lowland populations were no longer evident. Instead, we observed a faster development time in tadpoles originating from crayfish invaded sites, which, on average, metamorphosed 10 days before tadpoles from uninvaded sites. Amphibians show an exceptional variety of larval anti-predatory strategies, and their expression can strongly depend on the context they experience^23,34,39^. Rapid development can allow tadpoles to metamorphose earlier and thus reduce exposure to predators, a strategy particularly common when predation pressure is high^19,20,40^. Although our data do not allow us to directly determine whether invaded populations now develop faster than prior to the crayfish invasion, the shorter intrinsic development time of invaded populations compared with the uninvaded ones suggests that they underwent a rapid evolutionary response driven by the predation pressure imposed by the crayfish. This pattern is in agreement with recent evidence of rapid evolutionary changes in native species after biotic invasions^7,8,10,41,42^, even though we cannot fully exclude a role for epigenetic or maternal effects (but see Supplementary Note 3 and Supplementary Table 3).

Beyond the strong differences between invaded and uninvaded populations, we observed a plastic shift in tadpoles reared in the presence of the invasive crayfish, as these tadpoles exhibited a faster development. During rearing, exposed tadpoles perceived crayfish presence through both visual and chemical cues, which represent key signals allowing predator detection and modulation of anti-predator responses in aquatic species^43–46^. The faster development of exposed individuals indicates that tadpoles were able to recognize crayfish cues as a predatory threat, and thus trigger a plastic ontogenetic shift in response to perceived predation risk. Prey are generally able to identify their native predators^47,48^, but the recognition of non-native predators can be more challenging^49,50^. Responses to invaders can occur as a reaction to unknown cues, as a response to generic risk cues (e.g. to moving predators), or if they produce signals (e.g. visual and chemical cues) shared with similar or related native predators^51–56^. For instance, the response of the Italian agile frog to the invasive crayfish could arise because American red swamp crayfish releases cues similar to those of native European crayfish (*Austropotamobius pallipes*), which is a common predator of amphibian larvae and often occurs in syntopy with these frogs^57^.

Tadpoles from invaded populations, independently of being exposed to the crayfish or not, showed faster development comparable to the plastic response observed in those from uninvaded populations that were exposed to crayfish. These findings align with the hypothesis of a canalization of development time in invaded populations, and corroborates the idea that adaptation in development time may arise through the rapid fixation of genotypes exhibiting adaptive plasticity^32,58^. Conversely, the plastic response was generally weak in invaded, foothill populations. Before the invasion, foothill populations already showed a fast development (Fig. 2a), and it is possible that invaded populations in this region were already close to the physiological limit of the species, beyond which further development acceleration was not possible^59,60^.

Accelerating development can be an effective escape strategy from predators, but life-history theory predicts carry-over effects, with possible trade-offs between the benefits afforded in one trait and the consequences on other traits affecting fitness^61–63^. In organisms with complex life cycles, the decoupling between different life-history stages is generally incomplete and this can exacerbate the influence of early-development constraints on traits at later stages^22,64^. Yet, selection can favour compensatory mechanisms that partially counterbalance the negative impacts on subsequent stages. Compensatory growth during development is well-documented in amphibians and it allows them to optimize their growth trajectory under variable conditions^24,65–67^. In our study, anti-predatory responses to crayfish resulted in an acceleration of development and a short duration of the larval stage, which is generally associated with disadvantageous post-metamorphic traits, such as smaller body size and poorer locomotor performance (Fig. 3a–c). This can occur because of the reduced time devoted to the acquisition of trophic resources in fast-developing tadpoles^68^. However, we did not detect negative consequences of the developmental acceleration induced by the crayfish on froglet performance. When accounting for development time, tadpoles from invaded populations and those exposed to the crayfish showed improved post-metamorphic performance (Figs. 3d–i, 4) that allowed compensation to the acceleration in development. In other words, although these froglets metamorphosed earlier than those not exposed to the crayfish, they showed comparable performance (Table 2d–f). Amphibian development is influenced by the complex interplay between genetic and environmental drivers, and compensatory growth can limit the impact of suboptimal conditions on larvae, for instance through differential resource allocation or behavioural plasticity^23,24,66^. Mechanisms mitigating the costs of developmental plasticity, like compensatory growth, can be particularly favoured in species facing environmental variability or exposed to novel selective pressures^66^. In our study, compensatory growth seems to counteract the costs of developmental acceleration, thus limiting potential negative impacts on post-metamorphic fitness. Nevertheless, predicting the lifetime outcome of carry-over effects is complex, and impacts on traits not considered here (e.g. physiological traits) are possible^24^, therefore additional data are needed to evaluate the overall impacts across life-history stages.

Invasive predators have been one of the major determinants of extinctions in modern times^1,69^. When native populations persist, the selective force exerted by invasives can swiftly promote the evolution of life-history traits limiting exposure to predation^7,42^. Our study shows that invasive predators can pose selective pressures stronger than environmental gradients, driving rapid adaptive shifts in native species and obscuring pre-existing variation among populations exposed to divergent ecological pressures. Such adaptive responses can have complex impacts on multiple life-history stages, and could even produce suboptimal phenotypes in some habitats or under particular selective pressures^16,41^. Forecasting the impacts of invasive species is notoriously difficult, and is further complicated by the multifaceted adaptive responses of native species. Integrating the complexity of these responses is essential to evaluate how invasive species affect population dynamics, and to assess their long-term consequences.

## Methods

### Focal species and study area

The study area is in Lombardy (north-western Italy, ~45.5 N, 9.2 E), and includes the drainages of the Ticino (West) and Adda (East) rivers (Fig. 1a). This region is characterized by a rich hydrographic network and includes both agricultural and urban areas, mainly in the southern lowlands, and hilly relieves scattered with woodlands in the north. We focused on the Italian agile frog, which is endemic to the lowlands of northern Italy and adjacent areas. It is listed by the IUCN as vulnerable due to ongoing population declines caused by multiple factors, including habitat loss, fragmentation and invasive species^70^. Italian agile frogs breed in ponds and ditches within forest areas, and has a very narrow altitudinal range (from the sea level to ~400 m in the foothills of the Alps^36^). Egg-clutches are laid in March, and metamorphosis generally occurs in late spring-early summer (June–July). Temperature is the main driver of development rate in amphibians^31,71^ and previous studies have shown that lowland and foothill populations are exposed to different climatic regimes and show local adaptation to the extant climatic gradient^25^. We considered tadpoles originating from multiple foothill and lowland populations, and covering the whole altitudinal range of the species within the study area. To compare climatic regimes between foothill and lowland habitats, we calculated the mean spring temperature (March–June) for all the collection sites (see Fig. 1b) from the CHELSA climatic data set at a resolution of 30 arc-s (roughly 900 × 650 m within the study area; ref. ^72^). Mean spring temperature is only one of many climatic parameters. Nevertheless, a principal component analysis (PCA) performed on five climatic variables (mean temperature during March–June, mean annual temperature, annual seasonality of temperature, summed annual precipitation, and seasonality of precipitation) returned one single component explaining 95% of variation, that was positively related to all the temperature variables (in all pairwise correlations, *r* > 0.97) and negatively related to all the precipitation variables (in all the correlations, *r* ≤ −0.95). Moreover, we used data from local meteorological stations to test whether environmental conditions remained consistent during the 2003–2017 period (Supplementary Note 1 and Supplementary Table 1).

The American red-clawed crayfish is native to north-eastern Mexico and south-central USA and was first detected in Lombardy in the early 2000’s^33^. Since then the crayfish has successfully colonized a large number of wetlands, particularly in the south of the study area. Natural and human-mediated dispersal caused a northward spread of the crayfish, which has colonized many Italian agile frog breeding sites since 2004 (Fig. 1a). Even though the invasive crayfish is now widespread in the study area, its distribution remains scattered, as artificial and natural barriers prevented colonization of some isolated freshwater systems^38,73^. In the foothills the pattern of invasion is patchy, and uninvaded wetlands are intermixed with invaded areas^73,74^. The crayfish represents a major threat for the freshwater communities of invaded ecosystems^30,75^, and in the study region is causing the decline of several amphibian species^28^.

### Tadpole rearing before the crayfish invasion

In 2003, we reared tadpoles under common conditions to assess differences in development time between lowland and foothill populations. This experiment was performed just before the invasive crayfish colonized the study area. In early March, we collected egg-clutch fragments from five populations (three from foothill: AL CU, MZ; two from lowlands: TC, ZB; Fig. 1a). We sampled five clutches for each population and, after hatching, 10 tadpoles from each clutch were randomly selected and placed in containers filled with 1.5 l of aged tap water (total: 250 tadpoles reared). Tadpoles were maintained under common laboratory conditions (12-h light–dark cycles at constant temperature of 20 °C) and fed ad libitum with lettuce and rabbit pellets. Development time was calculated as the number of days between hatching and metamorphosis (Gosner’s stage 45; fully-developed forelimbs and almost complete reabsorption of the tail^76^). Tadpole survival until metamorphosis was similar between lowland and foothill populations (*χ*^2^_1_ = 0.084; *P* = 0.771). The results of the 2003 experiment have been published in a previous paper^25^, but we here re-analyse these data with updated statistical tools for a better comparison with the 2017 experiment.

### Tadpole rearing after the crayfish invasion

To assess the response of frogs to the selective pressure posed by the alien crayfish, we repeated the analysis of larval development 14 years after the onset of the invasion. In spring 2017, we collected 54 egg-clutch fragments from nine populations (4–12 clutches per population). We sampled the same populations analysed in 2003 (except for ZB, where the species suffered a local extinction) plus five additional populations (three from foothills: AS, GA, MT; two from lowland: A4, T3; see Fig. 1a) in order to increase sample size. Three of the foothill and all the lowland populations were invaded between 2004 and 2009, while the invasive crayfish remains absent in the other foothill populations (Fig. 1a). Sample size was similar across populations with different climatic regime or invasion status (Supplementary Table 4). Clutch fragments were housed in individual containers at outdoor temperature until hatching.

The 2017 rearing experiment was slightly different from the 2003 one, as it was designed to detect both differences in development time between populations (considering potential effects of both climatic regime and invasion status), and plastic responses to the exposure to the invasive crayfish. At Gosner’s stage 25, we randomly selected six tadpoles from each clutch (total: 324 tadpoles) and photographed them to measure starting size (tadpole total length: from the tip of the snout to the tail tip). Tadpoles of each clutch were divided into two groups of three tadpoles (hereafter triads). Triads were randomly assigned to one of two rearing treatments: absence vs. non-lethal presence of the crayfish. Tadpoles were reared in 0.8 l containers filled with aged tap water and clustered in six 70 × 48 cm tanks (hereafter blocks; 18 triads per block). In the crayfish treatment, tadpoles were reared in presence of one adult crayfish, which was separated from the rearing container with a plastic net. Therefore, tadpoles were constantly exposed to non-lethal visual and chemical cues released by the crayfish. Tadpoles in the control treatment were maintained under identical conditions, except for the absence of crayfish. Tadpoles were reared outdoors and the tanks were shaded to mimic natural conditions. Tadpoles were exposed to natural diel temperature fluctuations, but the average temperature experienced by larvae during development was very similar to the 2003 conditions (19.8 ± 0.5 °C; Supplementary Note 2). During rearing, half of the water in the experimental tanks was changed weekly and both tadpoles and crayfish were fed ad libitum with rabbit pellets and fish food. When reaching Gosner’s stage 42 (emergence of the first forelimb), we transferred tadpoles in small individual containers with 5 mm of water and moved them to the laboratory, where they completed metamorphosis. Each container was provided with a plastic staircase offering froglets the possibility to move out of water. Tadpole development time was calculated as time from hatching to Gosner’s stage 45^76^, as in the 2003 experiment. In 2017, the experiment lasted from the 14 March (first hatch) to the 14 August (last tadpole attained metamorphosis). Tadpole mortality was unrelated to climatic regime of origin (generalized linear mixed model: *χ*^2^_1_ = 1.291; *p* = 0.256), crayfish presence in the breeding sites (*χ*^2^_1_ = 0.218; *p* = 0.641) or rearing conditions (*χ*^2^_1_ = 0.275; *p* = 0.600).

### Post-metamorphic traits

To assess carry-over effects on post-metamorphic traits, we measured morphology and jumping performance on 110 newly-metamorphosed froglets reared during the 2017 experiment (Gosner’s stage 45, almost complete tail resorption^76^). Among these froglets, 66% were from foothill populations, 67% came from populations invaded by the crayfish and 60% were exposed to the crayfish during rearing. We considered two morphological traits that are known to affect survival and locomotory performance of froglets, body length and tibiofibula length^64,77^. We photographed froglets on graph paper and measured morphological parameters from photos using ImageJ^78^. Five froglets were excluded from measurements due to low quality of the pictures. To evaluate post-metamorphic locomotor capacity, jumping performance of each newly-metamorphosed froglet, was assessed during one jump session. Jump sessions were conducted in laboratory at room temperature, and froglets were tested immediately after taking them out from the containers where they completed metamorphosis. During the jump session, we placed each froglet on plastic graph paper and stimulated jump by gently pushing its back with a wooden wand. Each session consisted in three consecutive jumping trials per individual during which the distance covered with each single jump was measured with a ruler^64^. Two froglets were excluded from jumping trials due to a hind-limb malformation, which hampered their normal locomotion. The repeatability of individual jumping performance across the three trials was high (repeatability tested using the *rptR* package^79^: *R* = 0.62, 95% CI = 0.51–0.71, *p* < 0.001). As a measure of jumping performance we considered the maximum distance, since in frogs, maximum jumping ability is more strongly related to feeding and escape ability than average jumping length^80–82^.

### Statistical analyses

For both the 2003 and the 2017 experiments, we used linear mixed models to assess the factors affecting development time of tadpoles. In all models, development time was square root transformed to improve normality. For the 2003 data, we used climatic regime (foothill/lowland) as a fixed factor, and both population of origin and clutch identity as random factors. Furthermore, development time is known to be strongly affected by intraspecific competition^34^, and some tadpoles died during development. Therefore, we also included the number of tadpoles surviving until metamorphosis per container as a covariate.

We used the same models to analyse development time after crayfish invasion (in 2017). Beside climatic regime, these models included two additional fixed factors: invasion status (invaded/not invaded populations), and crayfish exposure during rearing (absence/presence of crayfish); rearing block was an additional random factor. Preliminary analyses including years since invasion rather than invasion status yielded identical results and slightly lower values of fit. Climatic regime was included as a fixed factor (foothill/lowland), but results are identical if we used the first component of a PCA performed on five climatic parameters (Supplementary Table 5). We also tested possible two-way interactions between invasion status, crayfish exposure and climatic regime. In 2017 all lowland populations were invaded, thus it was impossible testing the interaction between invasion status and climatic regime.

Tadpole development can be also affected by non-genetic maternal effects; egg provisioning is a major maternal effect in amphibians^83^. To confirm that our results are not biased by differences in egg provisioning, we repeated analyses by including tadpole starting size (a proxy of egg provisioning) as additional covariate into models. Starting size did not show a significant effect on development time either in 2003^25^ or in 2017 (see Supplementary Note 3 and Supplementary Table 3), supporting the robustness of our conclusions.

Mixed models were also used to test the effect of development time, crayfish exposure, invasion status and climatic regime on post-metamorphic traits of froglets from the 2017 experiment, and to evaluate relationships between tibiofibula length and jumping performance. We performed two separate analyses, one including and one excluding development time as a covariate. Preliminary tests did not detect significant interactions between independent variables. In all these models post-metamorphic trait values were log-transformed and we considered population of origin, clutch identity and rearing block as random factors.

Finally, we performed a structural equation modelling (SEM) to elucidate the complex relationships between fixed factors, development time, post-metamorphic morphological traits and jumping performance of the 2017 experiment. SEM is a statistical method based on multiple regressions, which allows testing of hypotheses regarding multiple causal relationships among predictors, and estimating their role in explaining the observed variation of the dependent variable^84^. We included as fixed factors the invasion status and crayfish exposure; the number of living siblings at Gosner’s stage 45 was an additional covariate representing larval density during development. We did not consider climatic regime, as its effects on tadpole development were marginal (Table 1b). In SEM analyses, just one clustering parameter can be included. We therefore built three separate SEMs using block, population of origin and clutch identity as clustering parameters. In the main results we present the SEM including block as clustering parameter, as this was the analysis showing the most conservative results. Results were highly consistent when using site of origin or clutch identity as clustering parameters (see Supplementary Table 2). We performed analyses in R environment (version 3.4.1), using *lmerTest*, *lme4* and *MuMIn* packages for linear mixed models and *lavaan* package for SEM analysis^84,85^. In all mixed models, sample size was not homogeneous among groups, thus the degrees of freedom were approximated and can be non-integers^85^. Furthermore, we used *visreg* package^86^ to generate conditional regression plots.

### Ethical statement

All the experiments were performed under the authorization of Italian Ministry for Environment (DPN/17391 and Prot. N. 3383/T-A31). After metamorphosis, all the froglets were released in their site of origin. Before releasing tadpoles, we treated them with Virkon S to clear the potential presence of pathogens and avoid risk of disease spreading^87,88^.

### Reporting summary

Further information on research design is available in the Nature Research Reporting Summary linked to this article.

## Supplementary information

Supplementary Information

Peer Review

Reporting Summary

## Data Availability

All the experimental data are available in the fisgshare repository with the identifier [10.6084/m9.figshare.12452600.v2]. All meteoclimatic data used are available on the websites of the regional network of the Regional Environmental Agency (www.arpalombardia.it) and of the CHELSA high-resolution climate data set (http://chelsa-climate.org/). Source data are provided with this paper.

## Source data

Source Data
