# Supplementary material for: Rapid adaptation to invasive predators overwhelms natural gradients of intraspecific variation
Source: Nat Commun. 2020 Jul 17;11:3608. doi: 10.1038/s41467-020-17406-y (PMC7368066; doi:10.1038/s41467-020-17406-y)
Supplement: Supplementary file 1 — Supplementary Information [file 41467_2020_17406_MOESM1_ESM.pdf]

Supplementary Information for

Rapid adaptation to invasive predators overwhelms natural gradients of intraspecific variation

Andrea Melotto, Raoul Manenti, Gentile Francesco Ficetola

This PDF file includes:
Supplementary Notes 1-3
Supplementary Fig. 1
Supplementary Tables 1-5

Supplementary Notes

Supplementary Note 1 - Comparison of climatic regime between 2003 and 2017

To confirm that the observed developmental changes were not caused by other typologies of environmental change (e.g. climate change), we assessed whether climatic conditions changed in the study area during the study period. In so doing, we obtained daily meteorological data (mean temperature and summed precipitation) for the period 2000-2017, from the regional network of the Regional Environmental Agency (www.arpalombardia.it). Two climatic stations were nearby lowland populations, while two stations were nearby foothill populations (Supplementary Fig. 1).

We used linear models to test whether spring temperature or spring precipitation changed during 2000-2017. Models included the station as a fixed factor and the interaction between station and year was used to evaluate whether climatic change was heterogeneous across sites. These linear models confirmed the climatic differences between lowland and foothill stations, with foothill population being significantly colder and with slightly more precipitations (Supplementary Table 1). Within this period, we did not detect a significant temporal trend for these climatic parameters, nor significant interactions between year and station, thus it is unlikely that climatic change determined major evolutionary shifts among populations.

Supplementary Note 2 - Comparison of rearing condition between years

In 2003, tadpole rearing was conducted in a laboratory at constant temperature of 20°C and mean development time (from hatching to metamorphosis) was 55.1 ± 0.4 days (range: 48 to 79 days). Conversely, in 2017, tadpoles were reared outdoor to better mimic growth under natural conditions. Rearing tanks were posed under canopy cover to provide tadpole tree shade and mimic Italian agile frog breeding sites. Tadpole development in 2017 was longer (average: 105.0 ± 1.4 days; range: 73 to 145 days) than in 2003.

Differences in development between 2003 and 2017 did not occur because of differences in average temperature during rearing. In fact, we obtained temperature data from the regional network of the Regional Environmental Agency (www.arpalombardia.it). The climatic station selected was located in the city of Milano, approx. 600 m from the garden where the experiment was conducted. During the period of 21 March – 3 July 2017 (i.e. 105 days from mean hatching date), average temperature experienced by tadpoles was 19.8 ± 0.5 °C (range: 10.7 to 29.1°C), therefore mean temperature experienced by tadpoles during 2003 and 2017 was nearly identical (20°C and 19.8 °C, respectively). However, in 2017, tadpoles were exposed to a mean diel temperature fluctuation (DTF) of 8.7°C. When keeping equal mean temperature, DTFs often cause a slowdown of development rate in amphibians (1, 2), thus the longer development occurring in 2017 was likely caused by the temperature fluctuations experienced by tadpoles, and not by differences in mean temperature.

Comparison with the literature showed that the development time observed in 2017 is very consistent with development time observed in nature. Observations performed in 2003 suggested that, in the field, the minimum development time was ~75 days (3); in 2017 the minimum development time was 73 days. Monographies on the biology of the Italian agile frog indicate that, in nature, average development time is 90-120 days (4). This matches our results, as in our study the average development time was 105 days.

Supplementary Note 3 - Assessing potential maternal effects: analysis on development time including starting size

Potential maternal effects on agile frog development time was assessed by including starting size in the preliminary analyses. Starting size was obtained by measuring tadpole total length at Gosner's stage 25 (as tadpoles do not feed until stage 25, tadpole length at this stage is strongly related to total egg provisioning (5)). While influence of maternal effects on development time before the crayfish invasion were already excluded (3), we checked for the influence of any maternal effects within the 2017 experiment by comparing models with or without considering tadpole starting size. We used starting size as a proxy of egg size because Italian agile frog eggs are strictly clumped in clutches and attempts to individually isolate the eggs would result in a high risk of damaging embryos (6, 7). Previous studies have seen that tadpole starting size provides adequate information on the impact of maternal effects on tadpole development (6). After hatching, tadpoles were kept under the same laboratory conditions (constant 18°C). At Gosner stage 25, tadpoles were photographed to measure starting size. We then repeated analyses using starting size as an additional covariate, to evaluate whether our results are affected by differences in egg-size related maternal effects. When we added starting size as covariate, all results remained unchanged, and we did not detect significant relationships between starting size and development time (Supplementary Table 3).

Supplementary Figure 1. Location of climatic stations used to assess climatic variation during the 2000-2017 period (stars). Green and yellow symbols are the foothill and lowland sampled populations (see Fig. 1 for details).

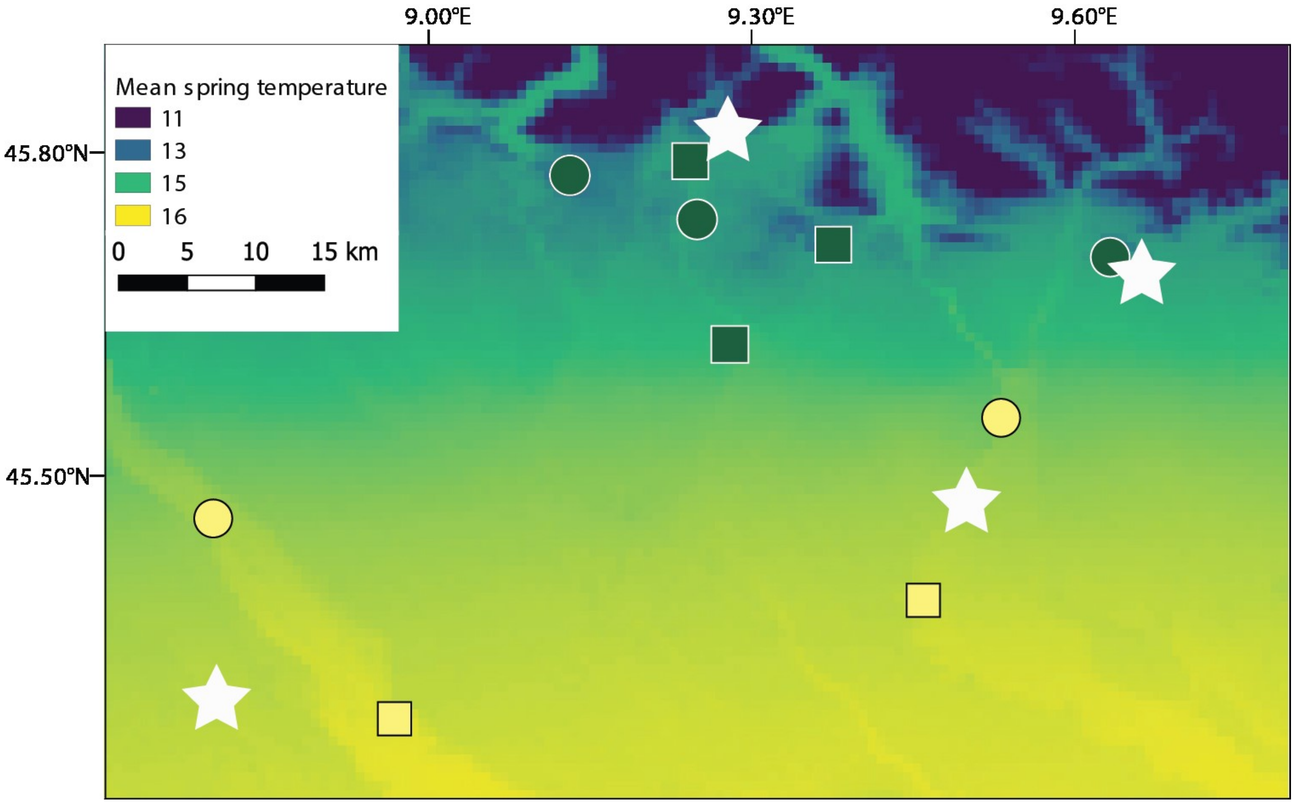

Supplementary Table 1. Assessment of climatic change during the period 2000-2017 in the study area for spring temperature and spring precipitation. Temperature and precipitation data were obtained from four meteorological stations, two in the lowland and two in the foothills (Supplementary Fig. 1). Significance values are calculated using two-sided F statistics, without multiple test corrections.

	Fixed effects	F	df	p
Mean spring temperature	Year	0.01	1,62	0.91
	Station	3.75	3,62	**0.015**
	Year * station	0.039	3,62	0.76
Total spring precipitation	Year	2.06	1,31	0.16
	Station	12.54	3,31	**< 0.001**
	Year * station	0.52	3,31	0.67

Supplementary Table 2. Results of structural equation models (SEM), considering different clustering parameters (block; population of origin and clutch identity). Coefficient of structural equation models including block (a), egg-clutch or site (c) as clustering parameters. Coefficient estimates, degrees of freedom, significance values, and determination coefficients are reported for each partial regression. Significant relationships are in bold. Significance values are calculated using two-sided z statistics, without multiple test corrections.

Supplementary Table 2a. SEM including block as clustering parameter

		Variances			
Dependent variable	Fixed effect	estimate	z	p	R^2
Jumping performance	Development time	0.035	0.691	0.490	0.598
	**Tibiofibula length**	**1.549**	**7.911**	**< 0.001**	
	Body length	-0.154	-0.413	0.680	
	Invasion status	-0.027	-1.074	0.283	
	Crayfish exposure	0.105	1.920	0.055	
	Number of siblings	-0.054	-1.589	0.112	
Tibiofibula length	**Development time**	**0.114**	**5.674**	**< 0.001**	0.305
	**Invasion status**	**0.090**	**3.745**	**< 0.001**	
	**Crayfish exposure**	**0.093**	**2.183**	**0.029**	
	**Number of siblings**	**-0.062**	**-3.096**	**0.002**	
Body length	**Development time**	**0.077**	**5.309**	**< 0.001**	0.294
	**Invasion status**	**0.045**	**2.229**	**0.026**	
	**Crayfish exposure**	**0.060**	**2.445**	**0.014**	
	**Number of siblings**	**-0.037**	**-3.713**	**< 0.001**	
Development time	**Invasion status**	**-0.550**	**-2.402**	**0.016**	0.213
	**Crayfish exposure**	**-0.630**	**-8.142**	**< 0.001**	
	**Number of siblings**	**0.417**	**3.002**	**0.003**	

		Covariances		
var. 1	var. 2	estimate	z	p
Tibiofibula length	**Body length**	**0.010**	**8.146**	**< 0.001**

Supplementary Table 2b. SEM including clutch as clustering parameter

Dependent variable	Fixed effect	Variances<br>estimate	z	p	R^2
Jumping performance	Development time	0.035	1.020	0.308	0.596
	**Tibiofibula length**	**1.549**	**5.731**	**0.001**	
	Body length	-0.154	-0.357	0.721	
	Invasion status	-0.027	-0.595	0.552	
	**Crayfish exposure**	**0.105**	**2.293**	**0.022**	
	Number of siblings	-0.054	-1.519	0.129	
Tibiofibula length	**Development time**	**0.114**	**6.281**	**0.001**	0.321
	**Invasion status**	**0.090**	**3.024**	**0.002**	
	**Crayfish exposure**	**0.093**	**2.713**	**0.007**	
	**Number of siblings**	**-0.062**	**-2.704**	**0.007**	
Body length	**Development time**	**0.077**	**7.047**	**0.001**	0.304
	**Invasion status**	**0.045**	**2.115**	**0.034**	
	**Crayfish exposure**	**0.060**	**2.777**	**0.005**	
	**Number of siblings**	**-0.037**	**-2.548**	**0.011**	
Development time	**Invasion status**	**-0.550**	**-3.679**	**0.001**	0.216
	**Crayfish exposure**	**-0.630**	**-3.698**	**0.001**	
	**Number of siblings**	**0.417**	**3.420**	**0.001**	

var. 1	var. 2	Covariances<br>estimate	z	p
Tibiofibula length	**Body length**	**0.010**	**6.867**	**< 0.001**

Supplementary Table 2c. SEM including population of origin as clustering parameter

Dependent variable	Fixed effect	Variances estimate	z	p	R^2
Jumping performance	Development time	0.035	1.399	0.162	0.596
	**Tibiofibula length**	**1.549**	**8.827**	**< 0.001**	
	Body length	-0.154	-0.522	0.602	
	Invasion status	-0.027	-0.760	0.447	
	**Crayfish exposure**	**0.105**	**4.775**	**< 0.001**	
	Number of siblings	-0.054	-1.782	0.075	
Tibiofibula length	**Development time**	**0.114**	**9.337**	**< 0.001**	0.321
	**Invasion status**	**0.090**	**5.106**	**< 0.001**	
	**Crayfish exposure**	**0.093**	**4.327**	**< 0.001**	
	**Number of siblings**	**-0.062**	**-3.640**	**< 0.001**	
Body length	**Development time**	**0.077**	**7.842**	**< 0.001**	0.304
	**Invasion status**	**0.045**	**2.059**	**0.039**	
	**Crayfish exposure**	**0.060**	**4.138**	**< 0.001**	
	**Number of siblings**	**-0.037**	**-3.496**	**< 0.001**	
Development time	**Invasion status**	**-0.550**	**-3.630**	**< 0.001**	0.216
	**Crayfish exposure**	**-0.630**	**-2.621**	**0.009**	
	**Number of siblings**	**0.417**	**4.253**	**< 0.001**	

var. 1	var. 2	Covariances estimate	z	p
Tibiofibula length	**Body length**	**0.010**	**7.302**	**< 0.001**

Supplementary Table 3. Factors determining development time of Italian agile frogs in the experiment conducted after crayfish invasion, considering starting size. Mixed models included the climatic regime (lowland vs. foothill), invasion status (crayfish invasion in the wetland of origin), and crayfish exposure (presence of crayfish in the container during rearing) as fixed factors. We also included the same interactions of the main model: invasion status x crayfish exposure; climatic regime x crayfish exposure. Besides, *n* of siblings in the container and starting size were included in all models, to take into account respectively of potential density and maternal effects on tadpole development time. Significant effects are in bold. Significance values are calculated using two-sided F statistics, without multiple test corrections.

Fixed effects	F	df	p
Climatic regime	0.46	1, 59.4	0.499
Invasion status	7.70	1, 51.7	**0.008**
Crayfish exposure	28.55	1, 160.5	**< 0.001**
N of siblings	8.41	1, 132.6	**0.004**
Starting size	0.10	1, 60.3	0.758
Invasion status x crayfish exposure	7.34	1, 158.3	**0.007**
Climatic regime x crayfish exposure	12. 53	1, 157.5	**< 0.001**

Supplementary Table 4. Average number of clutches per population. Comparison between foothill and lowland populations, and between invaded and uninvaded populations.

	***n* clutches**	
	**mean**	**se**
Foothill populations	5.4	1.5
Lowland populations	6.75	1.9
Invaded populations	6	1.3
Uninvaded populations	6	2.5

Supplementary Table 5. Factors determining development time of Italian agile frogs after crayfish invasion (mixed models, two-sided F statistics). In this analysis, climatic regime was represented by the first component of a principal component analysis (PCA) performed on five climatic factors (mean temperature during March-June; mean annual temperature; annual seasonality of temperature; summed annual precipitation; seasonality of precipitation). See Table 1 for details on the other independent variables.

Fixed effects	F	df	p
Climatic regime (PCA component)	0.34	1, 68.6	0.552
Invasion status	7.41	1, 63.2	**0.008**
Crayfish exposure	23.02	1, 163.5	**< 0.001**
N of siblings	5.99	1, 144.2	**0.016**
Invasion status x crayfish exposure	10.14	1, 166.3	**0.002**
Climatic regime x crayfish exposure	10.88	1, 167.5	**0.001**

Supplementary References

1. Kern P, Cramp RL, Franklin CE. Physiological responses of ectotherms to daily temperature variation. J Exp Biol. 2015;218(19):3068-76.
2. Arrighi JM, Lencer ES, Jukar A, Park D, Phillips PC, Kaplan RH. Daily temperature fluctuations unpredictably influence developmental rate and morphology at a critical early larval stage in a frog. BMC ecology. 2013;13(1):18.
3. Ficetola GF, De Bernardi F. Supplementation or in situ conservation? Evidence of local adaptation in the Italian agile frog *Rana latastei* and consequences for the management of populations. Animal Conservation forum: Cambridge University Press; 2005. p. 33-40.
4. Lanza B, Andreone F, Bologna MA, Corti C, Razzetti E. Amphibia: Edizioni Calderini; 2007.
5. Kaplan RH. Maternal influences on offspring development in the California newt, Taricha torosa. Copeia. 1985:1028-35.
6. Ficetola G, Visaggi B, Bonardi A, Padoa-Schioppa E, De Bernardi F. Starting size and tadpole performance in the frog Rana latastei. J Zool. 2011;284(1):15-20.
7. Ficetola GF, De Bernardi F. Offspring size and survival in the frog Rana latastei: from among-population to within-clutch variation. Biol J Linn Soc. 2009;97(4):845-53.
