# Supplementary material for: Rapid adaptation to invasive predators overwhelms natural gradients of intraspecific variation
Source: Nat Commun. 2020 Jul 17;11:3608. doi: 10.1038/s41467-020-17406-y (PMC7368066; doi:10.1038/s41467-020-17406-y)
Supplement: Supplementary file 3 — Reporting Summary [file 41467_2020_17406_MOESM3_ESM.pdf]

nature research

Corresponding author(s): Andrea Melotto

Last updated by author(s): Jun 16, 2020

Reporting Summary

Nature Research wishes to improve the reproducibility of the work that we publish. This form provides structure for consistency and transparency in reporting. For further information on Nature Research policies, see Authors & Referees and the Editorial Policy Checklist .

Statistics

For all statistical analyses, confirm that the following items are present in the figure legend, table legend, main text, or Methods section.

n/a	Confirmed	
☐	☒	The exact sample size (*n*) for each experimental group/condition, given as a discrete number and unit of measurement
☐	☒	A statement on whether measurements were taken from distinct samples or whether the same sample was measured repeatedly
☐	☒	The statistical test(s) used AND whether they are one- or two-sided<br>*Only common tests should be described solely by name; describe more complex techniques in the Methods section.*
☐	☒	A description of all covariates tested
☐	☒	A description of any assumptions or corrections, such as tests of normality and adjustment for multiple comparisons
☐	☒	A full description of the statistical parameters including central tendency (e.g. means) or other basic estimates (e.g. regression coefficient) AND variation (e.g. standard deviation) or associated estimates of uncertainty (e.g. confidence intervals)
☐	☒	For null hypothesis testing, the test statistic (e.g. F, t, r) with confidence intervals, effect sizes, degrees of freedom and P value noted<br>*Give P values as exact values whenever suitable.*
☒	☐	For Bayesian analysis, information on the choice of priors and Markov chain Monte Carlo settings
☒	☐	For hierarchical and complex designs, identification of the appropriate level for tests and full reporting of outcomes
☒	☐	Estimates of effect sizes (e.g. Cohen's *d*, Pearson's *r*), indicating how they were calculated

Our web collection on statistics for biologists contains articles on many of the points above.

Software and code

Policy information about availability of computer code

Data collection	No software was used for data collection
Data analysis	Analyses were performed under the R environment (version 3.4.1), using lmerTest (version 3.1-0), lme4 (version 1.1-21) and MuMIn (version 1.43.6) packages for linear mixed models, lavaan package (version 0.6-5) for SEM analysis, rptR (version 0.9.22) for reapetability test, and visreg package (2.5-0) to generate conditional regression plots. Detailed information on data analysis is available in the methods section. ImageJ software (https://imagej.net/, version 2.0.0-rc69/1.52p) was also used to collect body measurements of newly-metamorphosed froglets

For manuscripts utilizing custom algorithms or software that are central to the research but not yet described in published literature, software must be made available to editors/reviewers. We strongly encourage code deposition in a community repository (e.g. GitHub). See the Nature Research guidelines for submitting code & software for further information.

Data

Policy information about availability of data

All manuscripts must include a data availability statement. This statement should provide the following information, where applicable:

- Accession codes, unique identifiers, or web links for publicly available datasets
- A list of figures that have associated raw data
- A description of any restrictions on data availability

Data generated or analysed during this study are availabe in the figshare repository (https://doi.org/10.6084/m9.figshare.12452600.v1), or as regards meteorological data, these data are available at the regional network of the Regional Environmental Agency (www.arpalombardia.it) or can be downloaded from the CHELSA high-resolution climate data set (http://chelsa-climate.org/).

Field-specific reporting

Please select the one below that is the best fit for your research. If you are not sure, read the appropriate sections before making your selection.

☐ Life sciences ☐ Behavioural & social sciences ☒ Ecological, evolutionary & environmental sciences

For a reference copy of the document with all sections, see nature.com/documents/nr-reporting-summary-flat.pdf

Ecological, evolutionary & environmental sciences study design

All studies must disclose on these points even when the disclosure is negative.

Study description	The study compares development time in tadpoles of the Italian agile frog (Rana latastei) originating from populations exposed to contrasting climatic regime before (data collected in 2003) and after (2017) the arrival of the invasive American red swamp crayfish (Procambarus clarkii) in Northern Italy. Carry-over effects on fitness-related traits were also assessed in post-metamorphic froglets in the 2017 experimental rearing.<br>In 2003 experimental rearing tadpoles from three foothill populations and two lowland populations were reared in common laboratory conditions until metamorphosis (N = 250 individuals: five clutches per population, ten tadpoles per clutch). Development time variation between populations originating from different climatic regime was assessed through LMMs (N = 180 individuals surviving until metamorphosis).<br>In 2017 experimental rearing, tadpoles originated from six foothill and three lowland populations, which were invaded by the alien crayfish (six of them) or still uninvaded (three of them). Tadpoles (N = 324: 4-12 clutches per population, six tadpoles per clutch) were reared under two experimental conditions: absence and non-lethal presence of the invasive crayfish. Effects of population climatic regime (foothill vs lowland), presence of the invasive crayfish in the population of origin (invaded vs uninvaded) and experimental exposure to the crayfish during rearing (exposed vs unexposed), were tested on tadpole development time through LMMs (N = 169 individuals). In addition, two-way interaction between invasion status and crayfish exposure was assessed. Relations between development time and post-metamorphic morphological traits (body and tibiofibula length) and between tibiofibula length and jumping performance (calculated as maximum jumping distance on three replicate per individual) were also assessed through LMMs (N = 110 individuals).
Research sample	2003 experimental rearing: N = 250 Rana latastei tadpoles: five clutches per population, ten tadpoles per clutch. Factors influencing development time were assessed on 180 survived individuals suriving until metamorphosis. Survival did not significantly differ among populations originating from different climatic regimes.<br>2017 experimental rearing: N = 324 Rana latastei tadpoles: 4-12 clutches per population, six tadpoles per clutch. After metamorphosis, we assessed factors influencing development time (N = 169 individuals) and post-metamorphic traits (N = 110 individuals). Survival did not significantly differ among populations originating from different climatic regimes, or among populations invaded and uninvaded by the alien crayfish, or among treatments.<br>Selected populations origatinated from ten distinct breeding sites of Rana lastastei and were representative of the distribution of the focal species in Lombardy (Northern Italy). In both cases, the animals collected constitued a satisfying large number to perform robust analyses, while representing a neglegible impact on species populations.
Sampling strategy	During both rearing experiments (n 2003 and in 2017) we collected fragments from a total of 79 Rana latastei egg-clutches. Egg-clutch fragments constituted less than 30% of the entire egg-clutch and were obtained by carefully removing a mass of few eggs while preserving intact the rest of the egg-clutch. The collected egg-clutch fragments were transported to lab in buckets filled with pond water, where they were maintained until hatching. Sample size was selected to have statistical power larger than in historical analyses (explained at the beginning of par. Tadpole rearing after crayfish invasion)
Data collection	Tadpole development time were calculated as the number of days required to reach metamorphosis (Gosner's stage 45) from hatching. Morphological data were obtained by taking photos on graph paper of each individual at metamorphosis. Jumping performance was calculated as maximum jumping distance over three trials per individual. After each trial distance covered with a single jump was measured with a ruler. Animal measurents were performed using the image processing program ImageJ (https://imagej.net/, version 2.0.0-rc69/1.52p) Data were obtained by the three authors and students involved in the project.
Timing and spatial scale	As the Rana latastei reproduces and lay eggs in correspondence of the first heavy rains occurring in early spring, egg-clutches were sampled in this period (March- early April) both in 2003 and in 2017. Sampled populations were located in North Lombardy (approx. 45.5 N, 9.2 E) between the river basins of Adda and Ticino rivers, and covers the distributional range of the species in this region. The map of collection sites is available in Fig 1a
Data exclusions	Due to high mortality at metamorphosis, froglet morphological traits and jumping performance was assessed on 110 individuals only (2017 experimental rearing). Five froglets were excluded from measurements of post-metamorphic traits due to low quality of the pictures.
Reproducibility	All experimental setting is reproducible (i.e. rearing conditions, and collection of froglet jumping performance and morphological parameters). Nonetheless, as the invasive crayfish is actively spreading, we do not exclude the possibility that populations that were uninvaded at the time of data collection could become colonized by P. clarkii in the future.

Randomization	2003 experimental rearing: ten tadpoles were randomly selected from each egg-clutch and reared until metamorphosis in separete containers. Egg-clutch identity was included in the analyses as random factor.<br>2017 experimental rearing: six tadpoles from each clutch were randomly selected and divided into two groups of three individuals (hereafter triads). Each couple triad was randomly assigned to a different rearing treatment (non-lethal presence or absence of the invasive crayfish). Triads were randomly assigned to one out three rearing tanks per treatment where tadpole completed their development. Both egg-clutch and tank identity were included in the analyses as random factors.
Blinding	Operators were not blind during rearing or when performing tests, as to avoid mismatching errors all always individuals were identifiable by a unique code reporting their population of origin, clutch and treatment assignment.

Did the study involve field work? ☐ Yes ☒ No

Reporting for specific materials, systems and methods

We require information from authors about some types of materials, experimental systems and methods used in many studies. Here, indicate whether each material, system or method listed is relevant to your study. If you are not sure if a list item applies to your research, read the appropriate section before selecting a response.

Materials & experimental systems

n/a	Involved in the study
☒	☐ Antibodies
☒	☐ Eukaryotic cell lines
☒	☐ Palaeontology
☐	☒ Animals and other organisms
☒	☐ Human research participants
☒	☐ Clinical data

Methods

n/a	Involved in the study
☒	☐ ChIP-seq
☒	☐ Flow cytometry
☒	☐ MRI-based neuroimaging

Animals and other organisms

Policy information about studies involving animals; ARRIVE guidelines recommended for reporting animal research

Laboratory animals	The study did not involve laboratory animals.
Wild animals	For this study fragments from a total of 79 different egg-clutches of Italian agile frog (Rana latastei) were collected from 10 populations in Northern Italy across different years (2003 and 2017). Egg-clutch fragments constituted less than 30% of the entire egg-clutch and were obtained by carefully removing a mass of few eggs while preserving intact the rest of the egg-clutch. The collected egg-clutch fragments were transported to lab in buckets filled with pond water, where they were maintained until hatching. After rearing, metamorphosed froglets were released in their respective site of origin.
Field-collected samples	The study compares two experimental rearing performed on R. latastei tadpoles from different populations before (2003) and after (2017) the invasion of the American red swamp crayfish in Northern Italy.<br>2003 rearing protocol: tadpoles were maintained under common laboratory conditions (12-h light–dark cycles at constant temperature of 20° C) and fed ad libitum with lettuce and rabbit pellets. Tadpoles were reared in containers filled with 1.5 l of aged tap water (ten tadpoles from the same clutch per container; N =250). After rearing, metamorphosed froglets were released in their respective site of origin.<br>2017 rearing protocol: tadpoles (N = 324) were reared outdoor in 0.8 l containers (three individuals per container) which were hosted in six 70 x 48 cm tanks filled with 34 l of aged tap water; tanks were shaded to mimic natural conditions. During rearing individuals were exposed to two treatments: absence or non-lethal presence of the invasive crayfish, Procambarus clarkii. During rearing, half of the water in the experimental tanks was changed weekly and tadpoles were fed ad libitum with rabbit pellets and fish food. After rearing, metamorphosed froglets were released in their respective site of origin.
Ethics oversight	All the experiments were performed under the authorization of Italian Ministry for Environment (DPN/17391 and Prot. N. 3383/T-A31).

Note that full information on the approval of the study protocol must also be provided in the manuscript.
