# Supplementary material for: Rapid adaptation to invasive predators overwhelms natural gradients of intraspecific variation
Source: Nat Commun. 2020 Jul 17;11:3608. doi: 10.1038/s41467-020-17406-y (PMC7368066; doi:10.1038/s41467-020-17406-y)
Supplement: Supplementary file 2 — Peer Review [file 41467_2020_17406_MOESM2_ESM.pdf]

Reviewers' comments:

Reviewer #1 (Remarks to the Author):

I. Summary and strengths of the paper

This is a lovely and 'almost-complete' study of how adaptation to strong novel selection (from an introduced predatory crayfish), which has a) apparently resulted in the evolution over 14 years of a much earlier age of metamorphosis in a native Italian frog, has b) also thereby apparently eliminated the original gradient in that age between relatively low-lying warm climate subpopulations and higher elevation, and cooler climate populations that originally developed more quickly at any given temperature. Now that genetic distinction in age of metamorphosis has been lost, all subpopulations with crayfish metamorphose at a similar, younger age at a given temperature. The gradient of local adaptation to develop optimally with respect to the thermal environment has been lost.

That finding is relatively novel, and especially clear in this study system because you have published strong background work on the physiological ecology of the frog species, which in turn gives your interpretations substantial weight. In addition, the present 'chronosequence' study of before-and-after outcomes from the accidental 'experiment' of the crayfish introduction and spread tells a powerful story that is inherently memorable with keen heuristic value.

Moreover, you have taken the additional step of looking for possible costs to metamorphosing earlier. Suspecting such froglets might be less vigorous physically, you looked at body size and physical performance attributes. The size at metamorphosis is now smaller than before, hind limbs are shorter, and maximum jumping distance is shorter. On established first principles, it appears very likely that these altered attributes reduce both foraging and defensive performance.

These outcomes together make this study a good candidate to be a textbook example, one in which a system that would otherwise be a bit too complex for students and many researchers to fully internalize would instead be one whose lessons are readily retrievable and have lasting utility as touch points in broader discussions about the eco-evolutionary dynamics of biotic responses to anthropogenic change.

Draft Only

II. Chief questions and criticisms that are meant to contribute to potential revisions

1. Is there additional meaningful metapopulation detail that could be elaborated? How are native crayfish distributed within the study area? How much heterogeneity is there in terms of presence/absence and date of colonization by the predator of successive frog breeding ponds? Temporal heterogeneity would give you a more refined axis upon which to compare the degree of evolutionary change over time.

2. Is there a possible analysis that could act as an analog to an experimental control? Specifically, is there sufficient heterogeneity such that, e.g., comparisons of lowland and higher elevation frog sites WITHOUT crayfish could be compared to serve as a control in this study for other possible but unstudied sources of evolved acceleration of time to metamorphosis? As a related example, in the paper I led in 2005 on beak length evolution of a bug in response to an introduced host plant, it did not occur to me to check for change a congeneric bug in same study region that had not shifted onto the new host. I thought of that approach only on seeing another pioneering publication around that time showing rapid evolution of a smaller head size in snake that often attacked an introduced large-bodied toxic toad, and that used a comparison with a similar snake that did not attempt to prey on the deadly toad and kept its original head size as something of a control for the toad’s putative influence. Is there anything in your present data set that could serve this purpose, and thereby make your study more ‘complete’?

5. You regard the finding that exposed froglets are slightly larger at metamorphosis when development time is accounted (in the structure analysis) to be counterintuitive given your chief emphasized finding that age of metamorphosis is much reduced and predator-exposed froglets are altogether smaller. However my thought is that the analysis holds development time constant and therefore shows exposed froglets to be slightly larger per unit development time. If that interpretation is correct, the analysis suggests an additional phenotypic change— that growth is now faster during the abbreviated larval period as well. At present, you get lost in a bit of a miasma in the Results and Discussion about how multiple interacting factors can lead to unexpected outcomes that are apparently hard to explain. That feels like the weakest element of the paper. Are you perhaps not properly interpreting your analytical results on this point?

6. In addition, it *might* be of value to you to note that my own work on rapid adaptation to introduced host plants implies a couple of things. First, there was very poor relative performance at the time the new host is colonized, followed by rapid adaptation that came with an equally rapid loss of performance on the native host . That extreme tradeoff is reminiscent of your findings. Moreover, and complementary to your results, Cenzer (2016) shows loss of adaptation to the native host plant, apparently due to strong gene flow from subpopulations of the insect now well-adapted to the abundant introduced host plants. Citing this example in its entirety would be too much in your paper, but consider citing Cenzer (2016) as a complementary example a how anthopogenic adapatation can lead to complex unexpected problematic outcomes at the population level, perhaps noting the evidence of strong performance tradeoffs evident in the recent evolution of the derived subpopulation. The mechanism is different, but the topic addressed is basically the same.

III. Secondary points also worth addressing

1. The Discussion seems more anecdotal than vibrantly conclusive (style issue in part; see also comments 5 above). Perhaps overlong as well, and in that way more appropriate for a more specialized journal in your discipline.

2. Use of basic evolutionary terminology is rough regarding selection, evolution and adaptation. In the attached ms. pdf, I make a number of inline suggestions to address that concern.

3. Similarly, while the paper is generally quite well written, there are numerous instances in some sections in which the usage of English is imperfect. Those instances are also addressed in the attached edited pdf.

4. Note that the major inline comments on the attached pdf are generally redundant of those presented above.

Reviewer #2 (Remarks to the Author):

General comment:
The aim of this paper was to test how the presence of an invasive crayfish in Italy has affected development time (and subsequent fitness components) of Italian agile frog populations. The authors do this very nicely by comparing results of experiments performed in 2003, before the crayfish invaded the study area, with results of experiments performed more recently, after the crayfish has been established in the region for a while. The study is very interesting and relevant, but I believe that a much better and more detailed presentation and interpretation of the results is needed in order to support the authors' findings/conclusions. More details are provided in the comments below.

Draft Only

Major comments:
1. Even though I believe that the presence of P. clarkii was very likely the causal agent of change of development time in the studied populations of the agile frog, the authors do not report on any other possible confounding factors that could also be driving these alterations. For example, do the authors know if the climatic conditions in lowland and foothill populations have changed since their first experiments? Or were there any other alien species/predators potentially introduced into these water bodies? It would be important to be able to discard any possible effects imposed by these (or other) different factors in order to be absolutely certain that it is the presence of P. clarkii the sole factor driving the reported changes. A possibility that comes to mind is if e.g. the spring/summer average or maximum temperature in these areas has largely increased, or rainfall has largely decreased in the past years, which would cause ponds to dry faster and could also select for tadpoles development rates in both populations to become shorter (and similar).
In line with this thought, I believe it is fundamental that temperatures, rainfall and any other climatic variable that might influence the development time of the different populations studied is reported for both the 2003 and 2017 experiment.

2. Is there a specific reason why tadpoles were reared at 20°C in 2003, taking into account that the average spring temperature for these populations is around 14-16°C (at least in 2017)? Although this will not change the general outcome of the results, it can certainly affect the development time reported for each population. Also, given that in the 2017 experiment populations were reared outdoors, it would be important to have an idea of the average temperature that the tadpoles were exposed to, in order to understand if they are comparable to those from the experiments of 2003. In other words, if tadpoles were reared/exposed to very different temperatures in the different experimental years, it would be misleading to compare their development time, as the former can influence the time that tadpoles take to metamorphose.

3. Although I assume so, it is not entirely explicit in the manuscript if the authors calculated development time in the two experiments exactly in the same way. For the 2003 experiment, it is clearly stated that development time was calculated as the number of days between hatch and metamorphosis, while this is not stated in the protocol for the experiment in 2017 (which should be added around line 284). However, the legend of figure 2 states that development time has been calculated as the scaled days from Gosner stage 25 to metamorphosis. Does this mean that, for the statistical analyses of development time in the two experiments, the number of days between hatch and metamorphosis was used, but then for figure presentation the scaled days from Gosner stage 25 to metamorphosis were used instead? If this is the case, it should be made explicit in the manuscript, and a definition of 'scaled days' should also be added to the figure legend.

4. An important issue is that it is unclear in the methodology what exactly the number of replicates per population in the 2017 experiment is. The authors mention the use of 4-12 clutches per population, and the use of 6 tadpoles per clutch, so I assume that the number of replicates per population varies. In fact, I believe the authors actually acknowledge this in lines 333-334. Was there a specific reason for this to happen? To have a fixed number of replicates per population would have ensured equal representation of the different clutches/populations, would have been more in line with the 2003 experiment and is usually statistically desirable.

5. How long did the experiments last, i.e. how long were the tadpoles and crayfish kept in the laboratory? Because development time is never shown as an absolute value, it is not possible to estimate this. For example for the 2017 experiment, in case the tadpoles took quite a while to metamorphose, the crayfish surely had to be fed (regularly). If yes, it is important to know what were the crayfish fed with, e.g. conspecific tadpoles or other food items? This should be indicated somewhere in the methods, as it might impact on the type of visual and chemical cues that the tadpoles were exposed to.

6. Regarding jumping performance, it is not clear to me what the protocol was exactly. What do the

Draft Only

authors mean by a trial? Does this consist of a series of jumps or is it just one jump? I assume it is the former, but then is this defined by a certain time period, or by a certain number of jumps? In case a trial is simply one jump, I think to undertake only 3 jumps is quite a small number, as often the first jumps of froglets (after poked) are quite small and random, and only after a couple of jumps they become more regular and consistent. It seems unlikely to me that a froglet would jump its maximum jumping distance in only 3 jumps (especially in the lab). In any case, this protocol has to be better explained in the manuscript.

7. Another of my major concerns is about the way the results are presented and the conclusions that are drawn from them. For instance, Figure 2 should show results in much more detail. Instead of showing overall averages for different climatic regimes, invasion status and exposure treatment, I think it would be useful (and needed) to present graphs crossing the responses for all these different independent variables. This would make it much easier for the reader to quickly understand what has been found, e.g. what was the (change in) development time for the foothill, no crayfish presence, crayfish exposed populations, in comparison with crayfish presence and crayfish exposed ones, for instance. As a side note, I assume what is presented in Figure 2b, for example, is an average of foothill populations with and without crayfish, exposed or not, correct? Although I understand that all lowland populations had crayfish presence, it would be very relevant to show the average development time for both the foothill populations with and without crayfish, as it is fundamental to understand if populations still not colonised by crayfish maintained a similar development time as in the 2003 experiment, which would be expected in case crayfish presence is the only factor driving changes. For what I can see in Figure 2c, I assume that the development time in these latter populations actually became slower, and probably with no significant changes, which would make sense, but it is very important to show and acknowledge these results. In addition, as some of the populations where crayfish is present have been colonised in different years, it would also be very interesting to know if there are differences in development time between these different populations.

8. It is also important to show the differences in development time for the different populations under different climatic regimes and crayfish presence, exposed and not exposed to crayfish. As the authors do not show/consider any interaction terms in their statistical analyses, it is not possible to understand what is happening in these cases. If crayfish has imposed a canalised faster development time in populations with crayfish, then I believe that populations with crayfish, independently of being or not exposed to crayfish, should show this fast development. On the other hand, populations where crayfish is not present, can either show plasticity or not respond at all to exposure to crayfish. All these results should be presented and discussed.

9. As mentioned in the introduction, it is not surprising that there was a positive relationship between development time and morphology/jumping performance of froglets so, once again, I believe it would be of much more interest for the authors to show the differences between the different treatment combinations, instead of the overall results for all the animals combined. In fact, for the authors to be able to state that 'accelerated development following invasion leads to changes in morphology and jumping ability of frogs', they should show that this is not the case for populations that were never exposed to crayfish.
In line with this, in the methods section, it is important to indicate how many froglets from each population and treatment had their morphology measured and undertook jumping trials, in order to make sure that there was a similar representation from all of them.

Minor comments:
Line 74: I recommend replacing 'determines' by 'imposes'.
Line 80: Add 'pressure' after predation.
Lines 90-92, 100-109, 122-124: This text is part of the methodology, so there is no need to repeat it here – unless it is a requirement from the journal to summarise the methods in the Results section.
Line 138: add 'to', in 'exposed to the crayfish'.
Lines 171-172: 'with a consistent pattern across populations' – where can the reader see these

Draft Only

results?
Lines 178-179: Responses to invaders can also be triggered for other reasons, such as reaction to unknown smells of 'danger' or moving predators (given that here visual cues were also available), etc.
Line 181: Can you name which native crayfish species you mean?
Line 223: Correct the spelling of 'includes'.
Line 238: It is quite imprecise to state that P. clarkii is native to Western North America. Its native range is approximately the Northeastern of Mexico and Southcentral USA, please change accordingly.
Line 254: Just out of curiosity, was there a specific reason why the authors decided to calculate development time until Gosner stage 45, and not until stage 46, when metamorphosis is complete and the tail completely reabsorbed?
Line 266: Should be 2009, instead of 2008.
Line 272: Would be useful to state here that by body size it is meant tadpoles' body length.
Lines 275-276: It is not clear to me what the authors mean in this sentence – was each 0.8l container filled with water and then placed inside larger tanks?
Figure 2: I recommend showing all graphs in the same scale (e.g. -30 to 10), as it would make comparisons much easier.
Supplementary Material: It would be useful if the 'names' of the fixed effects in the tables shown here would be consistent with those used in the tables/figures of the manuscript.

Draft Only

Reviewer #3 (Remarks to the Author):

This is a very interesting paper, relating historically the interaction between two different selective pressures on anuran life history traits. The paper is well written, of general interest, and brings clearly the general message. My main questions refer to the way climate is characterized and statistically included in the analysis, as well as the variables controlled during jumping performance tests. Some additional minor questions and comments are also reported bellow:

Line 38: I think the statement "and to improve fitness" is unnecessary. Adaptation already implies improved fitness.

Line 45: Do the authors really mean comparing species? Or populations? Or both?

Line 69: "cold climate" seems a poor way to refer to a climate. Colder temperatures are just one aspect of climate, and temperatures may still vary seasonally... Can the authors characterize better the climates (or the climate) in the study area?

Line 235: is the mean temperature in spring enough or the best way to characterize temperature variation in these habitats?

Lines 292-294: Tests of jumping performance were performed at which temperature? Were the individuals hydrated? For how long were the individuals maintained at standard conditions before tests? Do the individuals still have a tail when tested? Did it vary between individuals and might have affected the results? Describe these methods better.
Additionally, Are 3 jumps enough to characterize individual variation? Report tests of repeatability.

Lines 300-301: I think the authors might justify better why they chose to insert climate as a factor with two categories instead of treating it as a continuous climatic variable, based on a PCA of climatic variables, for example.

RESPONSE TO THE COMMENTS OF REVIEWER 1

This is a lovely and 'almost-complete' study of how adaptation to strong novel selection (from an introduced predatory crayfish), which has a) apparently resulted in the evolution over 14 years of a much earlier age of metamorphosis in a native Italian frog […]

That finding is relatively novel, and especially clear in this study system because you have published strong background work on the physiological ecology of the frog species, which in turn gives your interpretations substantial weight. In addition, the present 'chronosequence' study of before-and-after outcomes from the accidental 'experiment' of the crayfish introduction and spread tells a powerful story that is inherently memorable with keen heuristic value.

Moreover, you have taken the additional step of looking for possible costs to metamorphosing earlier. Suspecting such froglets might be less vigorous physically, you looked at body size and physical performance attributes. The size at metamorphosis is now smaller than before, hind limbs are shorter, and maximum jumping distance is shorter. On established first principles, it appears very likely that these altered attributes reduce both foraging and defensive performance.

These outcomes together make this study a good candidate to be a textbook example, one in which a system that would otherwise be a bit too complex for students and many researchers to fully internalize would instead be one whose lessons are readily retrievable and have lasting utility as touch points in broader discussions about the eco-evolutionary dynamics of biotic responses to anthropogenic change.

Draft Only

We thank the reviewer for the positive comment and hereafter we report amendments we performed following reviewer's suggestions.

II. Chief questions and criticisms that are meant to contribute to potential revisions

1. *Is there additional meaningful metapopulation detail that could be elaborated? How are native crayfish distributed within the study area? How much heterogeneity is there in terms of presence/absence and date of colonization by the predator of successive frog breeding ponds? Temporal heterogeneity would give you a more refined axis upon which to compare the degree of evolutionary change over time*

1.R: Following the suggestion, we expanded the section pointing out that due to the occurrence of natural barriers, crayfish distribution in the study area is discontinuous. This results in a patchy pattern in the invasion of amphibian breeding sites, with few uninvaded systems within invaded areas (see Methods section, lines 286-289). We also cite recent work analyzing the situation within a metapopulation perspective (Manenti et al. 2020. Network-scale effects of invasive species on spatially-structured amphibian populations. Ecography 43, 119-127).

Populations showed some heterogeneity in colonization time by the predator (they were invaded 8-13 years before the second experiment), and we agree that taking into account such temporal heterogeneity might provide a more refined axis upon which to compare evolutionary change over time. Following this suggestion, and also following suggestions of Reviewer 2, we repeated analyses by including time since invasion (continuous variable) instead of invasion status (invaded vs uninvaded).

The model with invasion time was nearly identical to the model with invasion status, and showed a slightly higher Akaike's Information Criterion, suggesting that adding invasion time may not provide additional information (see below).

Analysis with invasion time as continuous variable:

Fixed effects	F	df	P	AIC
Climatic regime	0.30	1, 51.6	0.587	417.0
Invasion time (continuous variable)	10.14	1, 48.1	**0.003**	
Crayfish exposure	21.20	1, 163.9	**< 0.001**	
N of siblings	8.50	1, 147.0	**0.004**	
Invasion time*crayfish exposure	4.68	1, 159.9	**0.032**	
Climatic regime*crayfish exposure	10. 36	1, 162.1	**0.002**	

Analysis with invasion status as a fixed factor (invaded vs. uninvaded)

Fixed effects	F	df	P	AIC
Climatic regime	0.02	1, 52.3	0.879	416.5
Invasion status (fixed factor)	9.72	1, 51.6	**0.003**	
Crayfish exposure	30.13	1, 163.9	**< 0.001**	
N of siblings	7.06	1, 145.5	**0.009**	
Invasion status*crayfish exposure	6.30	1, 162.9	**0.013**	
Climatic regime*crayfish exposure	12.32	1, 161.4	**< 0.001**	

Draft Only

Moreover, to test for potential differences between populations early or recently invaded, we also repeated analyses considering "invasion status" as a fixed factor with three levels: invaded early (11-13 years ago; three populations); invaded recently (8-9 years ago, three populations); uninvaded (three populations). For this latter model, we used orthogonal contrasts (or planned comparisons, see Field et al., 2012) to perform the comparison between groups (contrast 1: uninvaded vs recently + early invaded populations; contrast 2: recently vs early invaded populations).

Field, A., Miles, J., & Field, Z. (2012). *Discovering statistics using R.*: Sage publications.

This new analysis yielded nearly identical results and showed a higher AIC value compared to the model including invasion status. In this analysis, contrasts confirmed the significant variation between uninvaded and invaded populations ($F_{1, 54.8}$ = 8.07; P = 0.006), but did not show any difference between early and recently invaded populations ($F_{1, 59.4}$ = 1. 17; P = 0.284). These results are in agreement with the ones presented in the manuscript, confirming that differences in invasion time are not strong enough to cause differences in development time, and corroborates the choice of invasion status as fixed variable to compare evolutionary change in our system. We expanded the methods section (lines 375-377) to clarify these points.

We did not include these additional analyses in the manuscript to avoid an excessively long methods section but, if the referee and the Editor believe this can improve the manuscript, we are ready to incorporate these analyses as supplementary materials.

Draft Only

Analysis with invasion stage as a fixed factor (early invaded, recently invaded, uninvaded)

Fixed effects	F	df	P	AIC
Climatic regime	0.07	2, 54.4	0.789	
Invasion stage	5.61	1, 55.65	**0.006**	
Crayfish exposure	26.25	1, 164.5	**< 0.001**	418.9
N of siblings	6.78	1, 142.3	**0.010**	
Invasion stage*crayfish exposure	3.06	2, 163.3	**0.049**	
Climatic regime*crayfish exposure	12.26	1, 160.6	**< 0.001**	

2. Is there a possible analysis that could act as an analog to an experimental control? Specifically, is there sufficient heterogeneity such that, e.g., comparisons of lowland and higher elevation frog sites WITHOUT crayfish could be compared to serve as a control in this study for other possible but unstudied sources of evolved acceleration of time to metamorphosis? As a related example, in the paper I led in 2005 on beak length evolution of a bug in response to an introduced host plant, it did not occur to me to check for change a congeneric bug in same study region that had not shifted onto the new host. I thought of that approach only on seeing another pioneering publication around that time showing rapid evolution of a smaller head size in snake that often attacked an introduced large-bodied toxic toad, and that used a comparison with a similar snake that did not attempt to prey on the deadly toad and kept its original head size as something of a control for the toad's putative influence. Is there anything in your present data set that could serve this purpose, and thereby make your study more 'complete'?

2.R: We agree with the reviewer that finding no corresponding alteration in development in a putative control species would undoubtedly corroborate our findings and reinforce our hypothesis on crayfish driving adaptation in development time of the Italian agile frog.

Analyses on congeneric species are an interesting idea. Unfortunately, this would require a species that is not preyed upon by *P. clarkii*. The non-native crayfish is an opportunistic predator with a wide trophic niche and in our system, and in our system all the anuran species are preyed upon by this invasive crayfish, as this predator can even feed on tadpoles of toxic species, such as toads (Gherardi et al., 2001; Renai & Gherardi 2004; Ficetola et al 2011). For this reason, we were not able to plan tests investigating the presence of similar trends in development time in other species not exposed to the crayfish predation. Moreover, comparisons of lowland and higher elevation frog sites WITHOUT crayfish are not possible in this region because today, unfortunately, all the lowland populations have been invaded by the crayfish.

Gherardi F, Renai B, Corti C (2001) Crayfish predation on tadpoles: A comparison between a native (*Austropotamobius pallipes*) and an alien species (*Procambarus clarkii*) Bulletin Français de la Pêche et de la Pisciculture:659-668.

Renai B, Gherardi F (2004) Predatory efficiency of crayfish: comparison between indigenous and non-indigenous species Biological Invasions 6:89-99.

Ficetola, G.F. et al. (2011). Early assessment of the impact of alien species: differential consequences of an invasive crayfish on adult and larval amphibians. Diversity and Distributions, 17(6), 1141-1151.

Draft Only

5. You regard the finding that exposed froglets are slightly larger at metamorphosis when development time is accounted (in the structure analysis) to be counterintuitive given your chief emphasized finding that age of metamorphosis is much reduced and predator-exposed froglets are altogether smaller. However my thought is that the analysis holds development time constant and therefore shows exposed froglets to be slightly larger per unit development time. If that interpretation is correct, the analysis suggests an additional phenotypic change— that growth is now faster during the abbreviated larval period as well. At present, you get lost in a bit of a miasma in the Results and Discussion about how multiple interacting factors can lead to unexpected outcomes that are apparently hard to explain. That feels like the weakest element of the paper. Are you perhaps not properly interpreting your analytical results on this point?

5.R: We thank the reviewer for this precious suggestion, which motivated us to further explore the apparent contrasting outcomes we observed and to perform new analyses.

We built new LMMs to assess the influence of the response to the crayfish on frog post-metamorphic traits (see Methods, lines 384-389 and Results, lines 146-154, 160-163). Model structure was the same as the one used for the analyses of development time, and included crayfish exposure, invasion status, climatic regime, and number of siblings as fixed factors. We built two separated models, one including development time as covariate, and one without development time. In both models, we found no negative influence of crayfish treatment or invasion status on post-metamorphic traits (see Table 2).

Conversely, when taking into account development time, analyses revealed that both crayfish exposure and origin from invaded populations had a positive effect on the most post-metamorphic traits (see Results, line 146-152; Table 2a-c and Figure 3), which highlighted the presence of additional phenotypic response, as

proposed by the reviewer. This relative increase in post-metamorphic condition observed in response to the crayfish, suggests that a plastic increase of growth rate (i.e. compensatory growth) is also present and counterbalances shifts induced by development acceleration. We expanded the discussion to highlight these plastic shifts and their role as potential compensatory mechanisms (lines 222-244). This new analysis greatly contributed disentangling and interpreting the multifaceted nature of the shift induced by of crayfish and significantly improved both the quality of the manuscript and clarity of the discussion.

*6. In addition, it *might* be of value to you to note that my own work on rapid adaptation to introduced host plants implies a couple of things. First, there was very poor relative performance at the time the new host is colonized, followed by rapid adaptation that came with an equally rapid loss of performance on the native host. That extreme tradeoff is reminiscent of your findings. Moreover, and complementary to your results, Cenzer (2016) shows loss of adaptation to the native host plant, apparently due to strong gene flow from subpopulations of the insect now well-adapted to the abundant introduced host plants. Citing this example in its entirety would be too much in your paper, but consider citing Cenzer (2016) as a complementary example a how anthopogenic adapatation can lead to complex unexpected problematic outcomes at the population level, perhaps noting the evidence of strong performance tradeoffs evident in the recent evolution of the derived subpopulation. The mechanism is different, but the topic addressed is basically the same.*

6.R: We thank the reviewer for indicating us these interesting works, which both provide a good example of rapid adaptive responses to invasive species and highlight the potential costs value of these shifts. We included both these studies in the introduction when discussing the complexity of responses to invasive species (line 38 and 47 and also cited Cenzer's study in the discussion when mentioning that adaptive shifts to novel selective pressures under can lead suboptimal performances when responding to other environmental pressures (line 250-252).

III. Secondary points also worth addressing

1. The Discussion seems more anecdotal than vibrantly conclusive (style issue in part; see also comments 5 above). Perhaps overlong as well, and in that way more appropriate for a more specialized journal in your discipline.

R: Following reviewer suggestion, and in the light of new analyses performed after the revision, we substantially modified the discussion, aiming to improve the clarity and incisiveness of our conclusion. The discussion has been partially expanded to address the comments raised by the three reviewers, still we tried to keep it as concise as possible.

2. Use of basic evolutionary terminology is rough regarding selection, evolution and adaptation. In the attached ms. pdf, I make a number of inline suggestions to address that concern.

R: We followed reviewer suggestion and modified text and terminology accordingly, where the text was not subject to other major changes.

Draft Only

3. Similarly, while the paper is generally quite well written, there are numerous instances in some sections in which the usage of English is imperfect. Those instances are also addressed in the attached edited pdf.

R: We thank the reviewer for the thoughtful revision and performed the changes suggested, where the text was not subject to other major changes.

4. Note that the major inline comments on the attached pdf are generally redundant of those presented above.

R: We performed all changes suggested and modified sections accordingly to the comments, see for instance lines 182-183, 205-208, regarding crayfish invasion, or post-metamorphic traits, line 141 and lines 146-163.

Finally, we remark that figure colors we chose were selected on a color-blind proof criterion, on the basis of palettes available at https://personal.sron.nl/~pault/

******

RESPONSE TO THE COMMENTS OF REVIEWER 2

The aim of this paper was to test how the presence of an invasive crayfish in Italy has affected development time (and subsequent fitness components) of Italian agile frog populations. The authors do this very nicely by comparing results of experiments performed in 2003, before the crayfish invaded the study area, with results of experiments performed more recently, after the crayfish has been established in the region for a while. The study is very interesting and relevant, but I believe that a much better and more detailed presentation and interpretation of the results is needed in order to support the authors' findings/conclusions […].

1. Even though I believe that the presence of P. clarkii was very likely the causal agent of change of development time in the studied populations of the agile frog, the authors do not report on any other possible confounding factors that could also be driving these alterations. For example, do the authors know if the climatic conditions in lowland and foothill populations have changed since their first experiments? Or were there any other alien species/predators potentially introduced into these water bodies? It would be important to be able to discard any possible effects imposed by these (or other) different factors in order to be absolutely certain that it is the presence of P. clarkii the sole factor driving the reported changes. A possibility that comes to mind is if e.g. the spring/summer average or maximum temperature in these areas has largely increased, or rainfall has largely decreased in the past years, which would cause ponds to dry faster and could also select for tadpoles development rates in both populations to become shorter (and similar).

In line with this thought, I believe it is fundamental that temperatures, rainfall and any other climatic variable that might influence the development time of the different populations studied is reported for both the 2003 and 2017 experiment.

1.R: We thank the reviewer for highlighting this point and we agree that it is important excluding environmental variation as potential driving factors of the development shift. Following the reviewer suggestion, we tested whether temperature and precipitation showed a significant trend during the period

Draft Only

2000- 2017. We obtained the daily spring temperature and precipitation from the Regional Environmental Agency and we used mixed models to assess their variation during this period (see supplementary text S1 and Table S1). LMMs showed no significant variation in temperature (P = 0.91) or precipitation regime (P = 0.16) has occurred over the fourteen-year period. Furthermore, climatic differences between lowland and foothill environments remained consistent along this period (Table S1). Thus, results suggest that between 2003 and 2017 no evident change in the climatic regime of the study area has occurred. To clarify these points we expanded the results section (lines 107-109 and see methods 280-281) and added the extended version of these new analyses in the supplementary results (Supplementary text S1; Figure S1; Table S1).

2. Is there a specific reason why tadpoles were reared at 20°C in 2003, taking into account that the average spring temperature for these populations is around 14-16°C (at least in 2017)? Although this will not change the general outcome of the results, it can certainly affect the development time reported for each population. Also, given that in the 2017 experiment populations were reared outdoors, it would be important to have an idea of the average temperature that the tadpoles were exposed to, in order to understand if they are comparable to those from the experiments of 2003. In other words, if tadpoles were reared/exposed to very different temperatures in the different experimental years, it would be misleading to compare their development time, as the former can influence the time that tadpoles take to metamorphose.

2.R: We agree with reviewer differences in temperature experienced during ontogeny have profound influence on tadpole development, and we assessed this issue accordingly. Rearing in 2003 was performed in a constant-temperature room, following a standard protocol (see Ficetola & De Bernardi 2005)

Unfortunately, in spring 2017 the facility used in 2003 was not available anymore, and we decided rearing tadpoles outdoor, which also allowed to better mimic conditions actually experienced by tadpoles in nature.

Following reviewer suggestion, we used daily meteorological data to calculate the mean temperature experienced by tadpoles reared under outdoor conditions in 2017 (see supplementary text S2). Mean daily temperature data were obtained through the Regional Environmental Agency, and were recorded from a climatic station located in close proximity to the garden where tadpoles were reared. The average temperature experienced in 2017 was 19.8 ± 0.5 °C. Thus, mean temperature experienced by tadpoles during the two experiments was extremely similar.

The main difference between the two experiments was that in 2017 tadpoles experienced also to diel temperature fluctuations. Diel temperature fluctuations often delay metamorphosis in amphibians, compared to constant temperature regimes (Arrighi et al., 2013; Kern et al., 2015). Because of these fluctuations, development time in 2017 was longer than the one observed in 2003 even though average temperature was very similar.

Finally, we agree that Fig. 1 shows a mean spring temperature of 15°C (i.e. lower than the 20°C observed in our study). This occurs because the figure displays temperature during spring (March-June), but hatch generally occur in mid-march (21 March in 2017) and metamorphosis generally occur in early July (average: 3 July in 2017). The temperature during the period 21 March – 3 July is a bit higher than the temperature during 1 March – 30 June.

To clarify these points, we expanded the methods and results section (lines 329-331 and 131-133), and we added a section of supplementary results (Supplementary text S2).

Arrighi et al. (2013). Daily temperature fluctuations unpredictably influence developmental rate and morphology at a critical early larval stage in a frog. *BMC ecology*, *13*(1), 18.

Draft Only

Kern et al. (2015). Physiological responses of ectotherms to daily temperature variation. *Journal of Experimental Biology*, *218*(19), 3068-3076.

3. Although I assume so, it is not entirely explicit in the manuscript if the authors calculated development time in the two experiments exactly in the same way. For the 2003 experiment, it is clearly stated that development time was calculated as the number of days between hatch and metamorphosis, while this is not stated in the protocol for the experiment in 2017 (which should be added around line 284). However, the legend of figure 2 states that development time has been calculated as the scaled days from Gosner stage 25 to metamorphosis. Does this mean that, for the statistical analyses of development time in the two experiments, the number of days between hatch and metamorphosis was used, but then for figure presentation the scaled days from Gosner stage 25 to metamorphosis were used instead? If this is the case, it should be made explicit in the manuscript, and a definition of 'scaled days' should also be added to the figure legend.

3.R: We thank the reviewer for underlining this discrepancy, caused by a mislabeling of the figure. Labels of preliminary analyses inadvertently remained in the script that produced the final plots. In both studies development time was calculated as days from hatching to metamorphosis (Gosner's stage 45) and results reported in the paper refer to hatching-metamorphosis development time. We amended figure labelling, and clarified in the methods section that also in 2017 development time was calculated as days from hatching to metamorphosis (lines 335-336). Finally, in accordance to reviewer suggestion (point 5), the figure now shows the effective number of days instead of scaled days.

Draft Only

4. An important issue is that it is unclear in the methodology what exactly the number of replicates per population in the 2017 experiment is. The authors mention the use of 4-12 clutches per population, and the use of 6 tadpoles per clutch, so I assume that the number of replicates per population varies. In fact, I believe the authors actually acknowledge this in lines 333-334. Was there a specific reason for this to happen? To have a fixed number of replicates per population would have ensured equal representation of the different clutches/populations, would have been more in line with the 2003 experiment and is usually statistically desirable.

4.R: We agree that, in principle, using the same number of clutches per population would be preferable. However, our experimental plan was constrained by several factors:

(1) the need of using clutches laid in the same days (to avoid clutches experiencing different conditions);

(2) we studied a threatened species that is declining and, in some population has limited abundance. For some population no more than 4 clutches laid the same day were detected.

(3) maximize sample size. In the 2003 experiment, we only used 23 clutches (3-5 per population). To improve statistical power, in 2017 we used a larger number of clutches (9 populations, 54 clutches). This increased sample size; as in some population less than 6 clutches were available we expanded sample size in other populations belonging to the same group. We remark that the average number of clutches was very similar between lowland and foothill populations (6.7 vs 5.4) and between invaded and uninvaded populations (6 vs 6) (Table S4). We expanded the methods (lines 314-315) and added Supplementary Table S4 to clarify these points.

(4) Finally, to avoid effects of unequal sample size on statistical analyses, in mixed models degrees of freedom were approximated using Satterthwaite's methods (lines 403-404).

5. How long did the experiments last, i.e. how long were the tadpoles and crayfish kept in the laboratory? Because development time is never shown as an absolute value, it is not possible to estimate this. For example for the 2017 experiment, in case the tadpoles took quite a while to metamorphose, the crayfish surely had to be fed (regularly). If yes, it is important to know what were the crayfish fed with, e.g. conspecific tadpoles or other food items? This should be indicated somewhere in the methods, as it might impact on the type of visual and chemical cues that the tadpoles were exposed to.

5.R: Following the reviewer suggestion, we modified Fig. 2 and we now show the absolute values of development time (number of days), rather than reporting scaled days. The exact dates are now reported in Supplementary text S2.

The crayfish were fed *ad libitum* with rabbit pellets and fish food; this is the same food used for tadpoles. The description of food is in the methods section, now lines 331-333.

Draft Only

6. Regarding jumping performance, it is not clear to me what the protocol was exactly. What do the authors mean by a trial? Does this consist of a series of jumps or is it just one jump? I assume it is the former, but then is this defined by a certain time period, or by a certain number of jumps? In case a trial is simply one jump, I think to undertake only 3 jumps is quite a small number, as often the first jumps of froglets (after poked) are quite small and random, and only after a couple of jumps they become more regular and consistent. It seems unlikely to me that a froglet would jump its maximum jumping distance in only 3 jumps (especially in the lab). In any case, this protocol has to be better explained in the manuscript.

6.R: Following the suggestions of both reviewer 2 and reviewer 3, we expanded the methods section improving the clarity of the protocol assessing jumping performance (lines 348-358). Three jumps were performed by each froglets during a single jumping session. We now refer to jumping session for the test, while each jump was considered a single trial. Metamorphs of this species are thin, and previous studies showed that after 3-4 jumps some froglets are exhausted and jumping performance decreases (Ficetola & De Bernardi, 2006 Animal Conservation).
We used repeatability analysis to confirm the robustness of our measure of jumping performance. This new analysis confirmed that the repeatability of individual jumping performance across the three trials was good (repeatability tested using the *rptR* package: R = 0.62, 95% CI = 0.51-0.71, P < 0.001). We reported results of this analysis in the Methods section (lines 356-358).

7. Another of my major concerns is about the way the results are presented and the conclusions that are drawn from them. For instance, Figure 2 should show results in much more detail. Instead of showing overall averages for different climatic regimes, invasion status and exposure treatment, I think it would be useful (and needed) to present graphs crossing the responses for all these different independent variables. This would make it much easier for the reader to quickly understand what has been found, e.g. what was the (change in) development time for the foothill, no crayfish presence, crayfish exposed populations, in comparison with crayfish presence and crayfish exposed ones, for instance. As a side note, I assume what is presented in Figure 2b, for example, is an average of foothill populations with and without crayfish, exposed or not, correct? Although I understand that all lowland populations had crayfish presence, it would be very relevant to show the average development time for both the foothill populations with and without

crayfish, as it is fundamental to understand if populations still not colonised by crayfish maintained a similar development time as in the 2003 experiment, which would be expected in case crayfish presence is the only factor driving changes. For what I can see in Figure 2c, I assume that the development time in these latter populations actually became slower, and probably with no significant changes, which would make sense, but it is very important to show and acknowledge these results.

8. It is also important to show the differences in development time for the different populations under different climatic regimes and crayfish presence, exposed and not exposed to crayfish. As the authors do not show/consider any interaction terms in their statistical analyses, it is not possible to understand what is happening in these cases. If crayfish has imposed a canalised faster development time in populations with crayfish, then I believe that populations with crayfish, independently of being or not exposed to crayfish, should show this fast development. On the other hand, populations where crayfish is not present, can either show plasticity or not respond at all to exposure to crayfish. All these results should be presented and discussed.

7.R: We are grateful to the reviewer for comments at point 7 and 8, as they stimulated us performing deeper analyses and finally resulted in a better understanding of our findings. We address these two points together.

Following the reviewer suggestion, we performed a new analysis (see methods line 375-377) also including the interaction crayfish exposure X invasion status, and the interaction climatic regime X crayfish exposure. As noticed by the reviewer, it was impossible to test climatic regime x invasion status interaction, as all the lowland populations in our study were invaded. Including the two interactions simultaneously revealed both of them had a significant effect on frog development time. Both tadpoles from invaded populations and tadpoles from lowlands reduced more their development time when exposed to the crayfish during rearing (result section, lines 125-129). We added a new figure describing this pattern (Fig 2e). These new analyses helped in disentangling crayfish induced effects on tadpole development and their variation among populations.

As shown in Fig 2e, these interactions indicate that foothill populations and uninvaded populations have a particularly strong response to exposure to the crayfish. These results provide further evidence that existing pattern of counter-gradient variation between populations exposed to different climatic regimes has been lost following the arrival of the crayfish (compare Fig 2a with Fig 2e). Moreover, the acceleration of development rate caused by crayfish exposure was similar to the differences in development rate between invaded and uninvaded populations, in agreement with the hypothesis of canalization of existing plasticity (Levis et al 2018; Levis & Pfenning, 2019). These new results are reported and discussed at lines 209-217.

Levis, N. A. et al. (2018). Morphological novelty emerges from pre-existing phenotypic plasticity. Nature ecology & evolution, 2(8), 1289-1297.

Levis, N. A., & Pfennig, D. W. (2019). In *Seminars in cell & developmental biology* (Vol. 88, pp. 80-90). Academic Press.

In addition, as some of the populations where crayfish is present have been colonised in different years, it would also be very interesting to know if there are differences in development time between these different populations.

Draft Only

Following this suggestion, we tested for differences between populations invaded in different periods. We did not find differences between populations invaded recently (8-9 years ago) and populations invaded 11-13 years ago. We added some details in the methods section (lines 372-373); see response to Reviewer 1 for additional details and results of statistical analyses.

9. As mentioned in the introduction, it is not surprising that there was a positive relationship between development time and morphology/jumping performance of froglets so, once again, I believe it would be of much more interest for the authors to show the differences between the different treatment combinations, instead of the overall results for all the animals combined. In fact, for the authors to be able to state that 'accelerated development following invasion leads to changes in morphology and jumping ability of frogs', they should show that this is not the case for populations that were never exposed to crayfish.

In line with this, in the methods section, it is important to indicate how many froglets from each population and treatment had their morphology measured and undertook jumping trials, in order to make sure that there was a similar representation from all of them.

Draft Only

9.R: Following the suggestions of reviewers 1 and 2, we further explored the relationships between crayfish-induced acceleration in development and post-metamorphic traits.

We built new LMMs to better assess the influence of the response to the crayfish on frog post-metamorphic traits (see Methods, lines 384-389 and Results, lines 146-154). Model structure was the same as the one used for the analyses of development time, and included crayfish exposure, invasion status, climatic regime, and number of siblings as fixed factors. We built two separated models, one including development time as covariate, and one without development time. In both models, we found no negative influence of crayfish treatment or invasion status on post-metamorphic traits.

Conversely, when taking into account development time, analyses revealed that both crayfish exposure and origin from invaded populations had a positive effect on the most post-metamorphic traits (see Results, line 146-150 and Figure 3d-i), which highlighted the presence of additional phenotypic response, as proposed by reviewer 1. This relative increase in post-metamorphic condition observed in response to the crayfish, suggests a plastic increase of growth rate (i.e. compensatory growth) is also present and counterbalances shifts induced by development acceleration. We expanded the discussion to highlight these plastic shifts and their role as potential compensatory mechanisms (lines 222-244). This new analysis greatly contributed disentangling and interpreting the multifaceted nature of the shift induced by of crayfish and significantly improved the quality of the manuscript and clarity of the discussion, and are in agreement with the pattern observed in structural equation models. We modified Fig 3 to better show this pattern.

Moreover, we added in the methods section the percentages of newly-metamorphosed froglets per each condition (foothill/lowland, invaded/uninvaded, exposed/not exposed). See lines 344-346.

Line 74: I recommend replacing 'determines' by 'imposes'.

Line 80: Add 'pressure' after predation.

R: We performed the requested changes.

Lines 90-92, 100-109, 122-124: This text is part of the methodology, so there is no need to repeat it here – unless it is a requirement from the journal to summarise the methods in the Results section.

R: We added these parts, both aiming to address the style of the journal and to contribute to contextualize the results in order to improve their clarity (the methods are at the end of the manuscript). However, if the editor thinks this contextualization is unnecessary, we are ready to remove it.

Line 138: add 'to', in 'exposed to the crayfish'.

R: Done

Lines 171-172: 'with a consistent pattern across populations' – where can the reader see these results?

R: After performing new analyses, we removed this sentence.

Lines 178-179: Responses to invaders can also be triggered for other reasons, such as reaction to unknown smells of 'danger' or moving predators (given that here visual cues were also available), etc.

R: We expanded the discussion to highlight this possibility (line 203-205).

Line 181: Can you name which native crayfish species you mean?

R: We referred to the European crayfish, *Austropotamobius pallipes*. We expanded this part, see lines 205-208.

Line 223: Correct the spelling of 'includes'.

R: Done

Line 238: It is quite imprecise to state that P. clarkii is native to Western North America. Its native range is approximately the Northeastern of Mexico and Southcentral USA, please change accordingly.

R: We amended this part as suggested. Now lines 282-283.

Line 254: Just out of curiosity, was there a specific reason why the authors decided to calculate development time until Gosner stage 45, and not until stage 46, when metamorphosis is complete and the tail completely reabsorbed?

R: This calculation was repeated in 2017 for homogeneity with the experiment performed in 2003

Line 266: Should be 2009, instead of 2008.

R: We thank the reviewer for underlining this mistake, amended.

Line 272: Would be useful to state here that by body size it is meant tadpoles' body length.

R: Done

Lines 275-276: It is not clear to me what the authors mean in this sentence – was each 0.8l container filled with water and then placed inside larger tanks?

R: Yes, each single container was placed inside the tank, and water level during the experiment was kept constant, so tadpoles were reared in in single chambers of the capacity of 0.8 liters. We modified the sentence to improve clarity, see lines 323-324.

Draft Only

Figure 2: I recommend showing all graphs in the same scale (e.g. -30 to 10), as it would make comparisons much easier.

R: We modified the figures showing the absolute development time (number of days). We used the same scale for figures comparing development time during the same year.

Supplementary Material: It would be useful if the 'names' of the fixed effects in the tables shown here would be consistent with those used in the tables/figures of the manuscript.

R: Following the suggestion, we uniformed fixed factor names throughout all article sections.

******

RESPONSE TO THE COMMENTS OF REVIEWER 3

This is a very interesting paper, relating historically the interaction between two different selective pressures on anuran life history traits. The paper is well written, of general interest, and brings clearly the general message. My main questions refer to the way climate is characterized and statistically included in the analysis, as well as the variables controlled during jumping performance tests. Some additional minor questions and comments are also reported bellow:

Line 38: I think the statement "and to improve fitness" is unnecessary. Adaptation already implies improved fitness.

R: Following reviewer suggestion, we removed this statement.

Line 45: Do the authors really mean comparing species? Or populations? Or both?

R: To improve clarity, we modified the sentence to "comparing species responses before and after the introduction…", now line 48.

Line 69: "cold climate" seems a poor way to refer to a climate. Colder temperatures are just one aspect of climate, and temperatures may still vary seasonally... Can the authors characterize better the climates (or the climate) in the study area?

Line 235: is the mean temperature in spring enough or the best way to characterize temperature variation in these habitats?

R: We addressed these two points together.

Temperature is the main driver of development rate in ectotherms, including amphibians; for all the amphibians, larvae develop more rapidly at warm temperatures (see e.g. pag. 124-126 in Wells, K.D., 2007. The ecology and behavior of amphibians. The University of Chicago Press, Chicago).

To improve clarity, we modified the sentence to "with lower temperature" (now line 73). Furthermore, we added a new section in the supplementary material (supplementary text S1, Table S1) where we show that

Draft Only

differences in temperature between lowland and foothill populations remained consistent in the period 2000-2017.

We also remark that, at this scale, all climatic variables are strongly correlated to altitude. To show this, we applied a PCA to five climatic variables (mean temperature during March-June; mean annual temperature; annual seasonality of temperature; summed annual precipitation; seasonality of precipitation). The first PCA component explained 95.4% of variation of these variables, and was positively related to all the temperature variables (in all pairwise correlations, $r > 0.97$) and negatively related to all the precipitation variables (in all the correlations, $r \leq -0.95$). Moreover, including in the model the first PCA component rather than climatic regime (foothill vs lowland) as fixed factor produces no substantial changes on the results (see supplementary Table S5). We added these details to the methods (lines 274-281, 373-375) and to the supplementary material (Table S5).

As reported above, mean we selected temperature of water is the parameter since this abiotic parameter characterizing differences among these habitats is the one also known to have the strongest influence on tadpole development. In the methods section we clarify the importance of temperature for amphibian development rate (line 268).

Lines 292-294: Tests of jumping performance were performed at which temperature? Were the individuals hydrated? For how long were the individuals maintained at standard conditions before tests? Do the individuals still have a tail when tested? Did it vary between individuals and might have affected the results? Describe these methods better.

Additionally, Are 3 jumps enough to characterize individual variation? Report tests of repeatability.

R: Following reviewer suggestion, we expanded the methods section describing jump sessions (see lines 348-358). Jump sessions were conducted in laboratory on hydrated tadpoles at room temperature (25°C). Individuals were transferred to the laboratory after reaching Gosner's stage 42 (first forelimb emergence) and were hosted in a single plastic container provided both with water and an area above water level (line 334-335) until Gosner stage 45. At this stage, tadpoles were transferred on graph paper and immediately tested. Jump session consisted in three consecutive jumps, which took no more than five minutes per individual. These test protocol unlikely caused strong water loss in froglets or differences in individual conditions.

Metamorphs of this species are thin, and previous studies showed that after 3-4 jumps froglets are exhausted and jumping performance decreases (Ficetola & De Bernardi, 2006 *Evolutionary Ecology*, *20*(2), 143-158). Conversely, with three trials, jumping performance is consistent across trials. Following the reviewer suggestions, we used repeatability analysis to confirm the robustness of our measure of jumping performance. This new analysis confirmed that the repeatability of individual jumping performance across the three trials was good (repeatability tested using the *rptR* package: R = 0.62, 95% CI = 0.51-0.71, P < 0.001), line 356-358.

Draft Only

Lines 300-301: I think the authors might justify better why they chose to insert climate as a factor with two categories instead of treating it as a continuous climatic variable, based on a PCA of climatic variables, for example.

R: We used foothill vs lowland because there is no overlap in temperature between these two areas (see Fig. 1b; in all the foothill sites the mean spring temperature is <15°C, while in all the lowland sites the mean spring temperature is >15°C) and for consistency with previous studies highlighting evolutionary and ecological differences between these groups of populations (Ficetola & De Bernardi 2005 Animal Conservation).

To confirm that this climatic definition did not affect our conclusions, we repeated analyses by using (PCA).

Specifically, we considered five climatic variables: mean temperature during March-June; mean annual temperature; annual seasonality of temperature; summed annual precipitation; seasonality of precipitation.

When we applied PCA to these five variables, the first PCA component explained 95.4% of variation, and was used as independent variable in subsequent analyses. The analysis performed with the first PCA component provided identical results as those obtained with the model including foothill/lowland factor (these results are reported supplementary Table S5 and we integrated the methods section, lines 274-281 and 373-375).

Draft Only

REVIEWERS' COMMENTS:

Reviewer #1 (Remarks to the Author):

I find the author's responses to my criticisms and suggestions to be comprehensive and thoughtful. The development of new statistical models to incorporate my concerns is a great approach. The great strength of the study and the significance of the findings are now much more evident due to the revisions made with respect to the comments of all the reviewers.

Scott Carroll

Reviewer #2 (Remarks to the Author):

Comments for the Authors:

The authors have addressed my comments in an appropriate manner, and I believe the manuscript is now much improved (also due to comments from the other reviewers).

The significant comment I still have relates to comment 2 from my previous review. Because Figures 2 (a) and (b) are now shown in days from hatching (instead of scaled days), we can actually see that, as the authors mention, development time in 2017 for both lowland and foothill populations was much longer than that observed in 2003. The authors justify this by stating that in 2017 tadpoles were exposed to diel temperature fluctuations, which often delay metamorphosis in amphibians. This might be true, but the fact that the difference in development time between the two years is so very large (almost doubling in 2017!), makes me wonder if there might be any other factor influencing this, other than the difference in experimental conditions, which introduces a whole other level of complexity to the manuscript. In the references/papers that the authors provide to sustain their explanation, the differences in development stage due to temperature fluctuations are of a much smaller scale. This also relates to my comment 7, where I state that 'it is fundamental to understand if populations still not colonised by crayfish maintained a similar development time as in the 2003 experiment, which would be expected in case crayfish presence is the only factor driving changes.' At the very least, I would suggest that the authors discuss this interesting finding under the Discussion of the manuscript. Also, it is very important to be clear throughout the text that invaded populations developed faster than non-invaded populations in 2017, but not than prior to crayfish invasion (in 2003). For example, lines 22-24 could be changed to 'Following the invasion, tadpoles from invaded populations began to develop at a faster rate than those from non-invaded populations, reaching the metamorphosis phase sooner to avoid predation.'

Some minor comments follow below:
1. Lines 107-109: I recommend slightly changing the sentence to something like 'Conversely, the local climate did not show any evident changes in temperature or precipitation during 2000-2017 (both $p > 0.15$), and the climatic differences existing between foothills and lowlands areas remained consistent during this period (Supplementary text S1; Table S1).
2. In Figure 2 (e) one can see that 3 of the foothill populations had development time between 100 and 110 days, and the other one around 120 days, so I don't understand how in Figure 2 (b) the average days to metamorphosis for all those 4 foothill populations is below 100 days. Shouldn't it be higher?
3. I believe the English language needs to be appropriately revised throughout the manuscript (also in the Supplementary Materials) to ensure a high quality final manuscript.

Reviewer #3 (Remarks to the Author):

Draft Only

The authors did a very good job answering questions from all the reviewers, conducting additional analyses and improving learity and objectivess of the manuscript. I am really satisfyied with this version of the manuscript, which is much better than the first version.

Draft Only

DETAILED RESPONSE TO THE COMMENTS OF REVIEWER 2

The authors have addressed my comments in an appropriate manner, and I believe the manuscript is now much improved (also due to comments from the other reviewers).

Many thanks for the positive comments and for the insightful suggestions.

The significant comment I still have relates to comment 2 from my previous review. Because Figures 2 (a) and (b) are now shown in days from hatching (instead of scaled days), we can actually see that, as the authors mention, development time in 2017 for both lowland and foothill populations was much longer than that observed in 2003. The authors justify this by stating that in 2017 tadpoles were exposed to diel temperature fluctuations, which often delay metamorphosis in amphibians. This might be true, but the fact that the difference in development time between the two years is so very large (almost doubling in 2017!), makes me wonder if there might be any other factor influencing this, other than the difference in experimental conditions, which introduces a whole other level of complexity to the manuscript. In the references/papers that the authors provide to sustain their explanation, the differences in development stage due to temperature fluctuations are of a much smaller scale. This also relates to my comment 7, where I state that 'it is fundamental to understand if populations still not colonised by crayfish maintained a similar development time as in the 2003 experiment, which would be expected in case crayfish presence is the only factor driving changes.' At the very least, I would suggest that the authors discuss this interesting finding under the Discussion of the manuscript. Also, it is very important to be clear throughout the text that invaded populations developed faster than non-invaded populations in 2017, but not than prior to crayfish invasion (in 2003). For example, lines 22-24 could be changed to 'Following the invasion, tadpoles from invaded populations began to develop at a faster rate than those from non-invaded populations, reaching the metamorphosis phase sooner to avoid predation.

Draft Only

Following the suggestions by the Editor and by the Reviewer, we expanded the main text and the supplementary material to clarify that the development time observed in 2017 is very similar with development time observed in nature. Actually, the development time observed during the 2003 experiment was significantly faster than the development observed during the same year in nature (this observation was also reported at pag. 36 of Ficetola & De Bernardi 2005). Observations performed in 2003 suggested that, in the field, the minimum development time was ~75 days (Ficetola and De Bernardi 2005); in 2017 the minimum development time was 73 days. Monographies on the biology of *R. latastei* indicate that, in nature, average development time is 90-120 days (Lanza et al. 2007). This matches our results, as in our study the average development time was 105 days. This supports the idea that diel temperature is a main driver of these differences. Nevertheless, we highlight that other potential causes of these differences (e.g. other differences in experimental settings; unrecorded evolutionary processes) cannot be excluded.

The additional details are added in the main text (lines 132-134) and in the Supplementary Results (pag. 2, second paragraph).

Furthermore, we modified the text (abstract, main results the discussion) accordingly to reviewer suggestions. We made it explicit that invaded populations developed faster than non-invaded populations in 2017, but not than prior to crayfish (lines 21-22 and 119-120). Then, we highlighted that our data do not allow testing whether invaded populations now develop faster than prior to crayfish invasion, because differences in development time between 2003 and 2017 (due to diel variation of temperature, or other potential, unidentified causes) hampered this comparison, see lines 188-189.

Some minor comments follow below:

1. Lines 107-109: I recommend slightly changing the sentence to something like 'Conversely, the local climate did not show any evident changes in temperature or precipitation during 2000-2017 (both $p > 0.15$), and the climatic differences existing between foothills and lowlands areas remained consistent during this period (Supplementary text S1; Table S1).

2. In Figure 2 (e) one can see that 3 of the foothill populations had development time between 100 and 110 days, and the other one around 120 days, so I don't understand how in Figure 2 (b) the average days to metamorphosis for all those 4 foothill populations is below 100 days. Shouldn't it be higher?

3. I believe the English language needs to be appropriately revised throughout the manuscript (also in the Supplementary Materials) to ensure a high quality final manuscript.

We performed the modifications suggested by the reviewer. We expanded the figure legend following the journal's guidelines to clarify what is shown by the plots. The manuscript has been reviewed by a native English speaker (Laura Pollock, associate professor at the McGill University)

Draft Only
